# GC-MS-Based Metabolomics Provides Insights into the Biochemical Peculiarity of Seven Brown Algal Species of the Order Fucales

**DOI:** 10.3390/md24070227

**Published:** 2026-06-29

**Authors:** Elena Tarakhovskaya, Ekaterina Gulk, Bochao Yang, Paula Schliebe, Susan Billig, Claudia Wiesner

**Affiliations:** 1 Department of Plant Physiology and Biochemistry, Faculty of Biology, Saint Petersburg State University, Saint Petersburg 199034, Russia; kategulk@gmail.com; 2 Vavilov Institute of General Genetics, Saint Petersburg Branch, Russian Academy of Science, Saint Petersburg 199034, Russia; 3 Institute of Analytical Chemistry, Faculty of Chemistry, Leipzig University, 04103 Leipzig, Germany; yangbochao54@gmail.com (B.Y.); paula.schliebe@bbz.uni-leipzig.de (P.S.); billig@uni-leipzig.de (S.B.); 4 Biotechnological-Biomedical Centre (BBZ), Leipzig University, 04103 Leipzig, Germany; 5 Guest Science Programme, Biologische Anstalt Helgoland, Alfred-Wegener-Institut Helmholtz-Zentrum für Polar-und Meeresforschung, 27498 Helgoland, Germany; 6 German Centre for Integrative Biodiversity Research (iDiv) Halle-Jena-Leipzig, 04103 Leipzig, Germany

**Keywords:** brown algae, metabolomics, phlorotannins, polyols, citric acid, *Fucus*, *Ascophyllum*, *Sargassum*, *Pelvetia*

## Abstract

Brown algae are important primary producers in coastal ecosystems, where they provide habitat and food for numerous marine species. For humans, they provide raw materials (food, animal feed, and ingredients for pharmaceuticals and cosmetics) as well as ecosystem services such as coastal protection and carbon sequestration. The molecular characterization of brown algae is necessary to understand their role in ecosystems, their biochemical resources, and responses to environmental stresses—knowledge that is crucial for the sustainable use and biotechnological applications of seaweed. Within this context, we analyzed more than 300 primary and secondary metabolites by gas chromatography–mass spectrometry to elucidate the metabolic profiles of seven habitat-forming species of brown algae in the arctic and temperate seas. Metabolite profiles were discussed considering physiological and ecological characteristics of the different algae, thus revealing the taxon-specific biochemical signatures and metabolite patterns contributing to seaweed adaptation to their typical habitats. Three important groups of metabolites representing polyols, phenolic compounds, and organic acids, were analyzed and discussed in more detail. Our study revealed metabolic diversity of species from the same order and genus, thereby indicating a very distinct regulation at the molecular level to meet metabolic needs of the habitat. The knowledge of different compositions of algal extracts can be used to develop specialized applications for humans in cosmetic, medical, or nutritional sectors.

## 1. Introduction

Biochemical studies on marine macroalgae are currently highly relevant as these organisms find broad practical application in the modern world. Representatives of different taxonomic groups of seaweed are used as sources of essential minerals and numerous organic compounds extensively exploited by humans in the food and feed industries, medicine, agriculture, cosmetic production, etc. [1,2]. Among seaweed, brown algae (Phaeophyceae) are of special interest for several reasons. They are the most abundant group of macrophytes in cold and temperate seas where they contribute substantially to the global productivity and biogeochemical cycling in marine ecosystems. Brown algae with large thalli, such as those belonging to Fucales and Laminariales, are key species in intertidal and subtidal ecosystems. They form specific habitats for marine invertebrates, juvenile fish, and epiphytic algae by structuring the underwater environment, providing food, substratum for settlement, and shelter from excess light and desiccation during low tides, excreting their metabolites into water, etc. [3]. Moreover, laminarialean and, especially, fucalean algae are model seaweeds studied for valuable specific metabolites and their biological activity [4]. Numerous compounds exhibiting anti-inflammatory, antithrombotic, anti-oxidative, anticancer, antiviral, and antimicrobial activity have already been isolated from various species of Fucales [5,6].

Investigation of biochemical composition and screening for bioactive metabolites in the brown algae requires an adequate methodological approach. Among the methods used for this purpose, gas chromatography–mass spectrometry (GC-MS)-based metabolomics is one of the most informative and robust tools allowing for the simultaneous analysis of hundreds of low-molecular-weight compounds of various chemical natures, making it the method of choice for non-targeted characterization of complex biological samples [7,8,9,10,11]. Electron impact ionization produces highly specific fragmentation patterns, with unrivaled information for compound identification. Consequently, the approach can be well used to discover new valuable compounds for practical use as well as for a comprehensive analysis of biologically active extracts and preparations of natural origin, which is necessary to clarify their chemical composition. A comparison of the metabolite profiles of different organisms can serve as a basis for chemical taxonomy and as a useful complement to molecular data for phylogenetic studies and species identification [10,12,13]. Similarly, chemotaxonomic information can be used to predict the biochemical composition of organisms that have not yet been analyzed and to assess their potential practical relevance or their likely responses to environmental changes.

Our research focuses on detailed study and comparison of metabolite profiles; here, we present results on seven brown algal species of the order Fucales, typical for the cold and temperate seas of the North Atlantic. To date, metabolomics of seaweed is not yet one of the well-developed fields of plant physiology, and fucalean algae has been studied very unevenly from this perspective. In fact, only the metabolite profile of *Fucus vesiculosus* has been relatively well characterized [8,14,15,16], while the metabolomes of other fucoids have been addressed only in single works [17,18,19,20,21] or have not yet been studied. Besides the general lack of detailed biochemical studies on fucalean algae, most of the available reports focus exclusively on the dominating metabolites and/or compounds exhibiting various biological activities, such as fatty acids, sterols, and phlorotannins (e.g., [1,15,16,18,19,22,23,24]).

To our knowledge, this study is the first attempt at a detailed comparative analysis of low-molecular-weight metabolite profiles of several representatives of the order Fucales. We aim to explore general characteristics of the fucalean metabolome and to identify taxon-specific biochemical signatures and metabolite patterns that may contribute to algal adaptation to different habitats and environmental conditions.

## 2. Results

### 2.1. General Description of Seaweed Metabolite Profiles

GC-MS-based metabolite profiling of seven fucalean algae revealed 334 individual trimethylsilyl (TMS) and methoxime (MEOX)/TMS derivatives (Table S1). Among them, 160 compounds were identified by retention indices (RI), mass spectral similarity, and co-elution with authentic standards, whereas 93 other metabolites were tentatively annotated to specific chemical classes based on their RI values and mass spectral similarity to MS library data (NIST, GMD, and in house spectral libraries ASL). Some of the compounds (amino acids, sugars) were represented by several derivatives resulting from different contributions of MEOX and TMS groups or syn/anti isomerism of sugar TMS-methoximes. The overall number of identified or structurally annotated metabolites was 253, represented by 78 carbohydrates (mono-, di- and trisaccharides, polyols, sugar phosphates and sugar acids), 32 amino acids and their derivatives, including several non-proteinogenic amino acids (pipecolic acid, α-aminoadipic acid, β-alanine, γ-aminobutyric acid, etc.), 23 organic acids (tricarboxylic acid (TCA) cycle intermediates, glycolic, benzoic, 2-methylmalic acids, etc.), 40 metabolites related to lipids (fatty acids and their derivatives, fatty alcohols, squalene), 50 phenolic compounds (phloroglucinol and its oligomers, homogentisic acid, tetrahydroxybenzene, etc.), 14 nitrogenous bases, nucleosides and their derivatives, four amines and a miscellaneous group of metabolites including fucosterol, tocopherols, ascorbic acid, urea, etc. (Table S1). Another 64 analytes could not be unambiguously annotated and were marked as unknowns.

**Figure 1:**
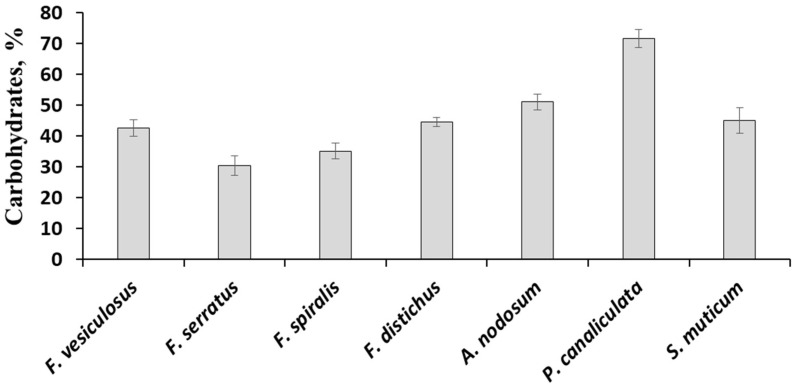
Relative intensity of carbohydrate-related signals in the TICs of the methanolic extracts of seven fucalean algae. Bars represent the means ± SD.

In general, metabolomic profiles of all studied algae were dominated by carbohydrates, with mannitol as the most abundant compound. The other major carbohydrates were genus-specific, e.g., volemitol and glycerol in *P. canaliculata*, polyols RI 2841, RI 3547, and glucose in *A. nodosum*, erythronic acid and isofloridoside in *S. muticum*. Overall, carbohydrate-related signals accounted for 30–71% of the total ion current (TIC) of the algal extracts, with the lowest values found in *F. serratus* and the highest in *P. canaliculata* (Figure 1). The other relatively abundant metabolites were fucosterol (all studied algae), citric acid (representatives of Fucaceae), glutamic acid (*Fucus* spp.), and alanine (*Fucus* spp. and *S. muticum*).

### 2.2. Comparison of Metabolite Profiles of Seven Fucalean Algae

**Figure 2:**
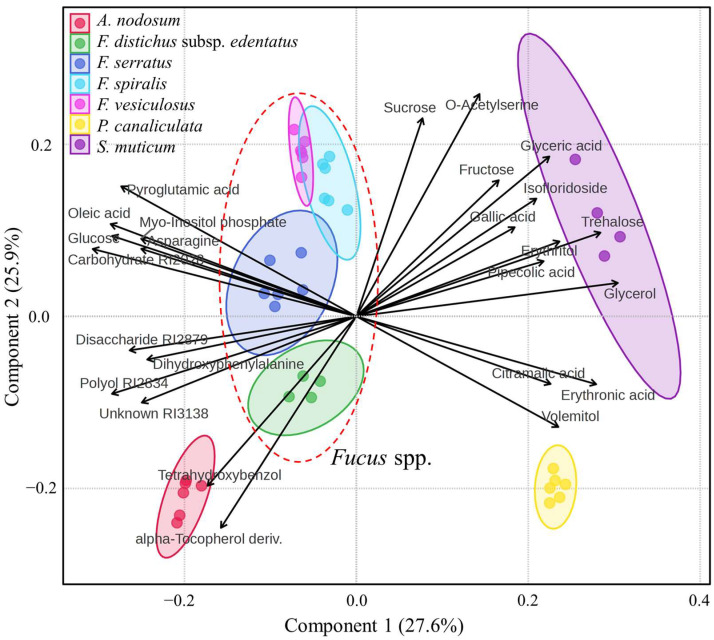
Biplot representing the sample scores and compounds with the highest loadings for the first two components derived from PLS-DA of the relative metabolite concentrations in the thalli of seven representatives of the order Fucales.

First, we used a partial least squares discriminant analysis (PLS-DA) to assess general similarities and differences in the metabolite profiles of the studied fucalean algae. Approximately 70% of the total variance was explained by the first three components (Figure S1), of which the first two covering 53.5% showed a clear clustering of the samples corresponding to various seaweed species (Figure 2).

Component 1 divides all samples into three distinct groups, where the first group is represented by *A. nodosum*, the second one includes four *Fucus* species, while the third one combines the samples of *P. canaliculata* and *S. muticum*. Analysis of the loadings showed that the metabolites contributing most to component 1 are predominantly carbohydrates (glycerol, mannitol, volemitol, trehalose, glucose, etc.) and amino acids (asparagine, glutamine, pyroglutamic acid, etc.). The other compounds with relatively high loadings include organic acids (α-ketoglutaric, citramalic, and glyceric acids), oleic acid and its derivative 2-oleoylglycerol, α-tocopherol derivative, and two phenolic compounds. Most carbohydrates, pipecolic, citramalic, and glyceric acids exhibited a higher relative abundance in the extracts of *P. canaliculata* and *S. muticum*, whereas *A. nodosum* and *Fucus* samples were enriched with amino acids, fatty acids, α-ketoglutarate, dihydroxyphenylalanine, and α-tocopherol derivative (Figure 2, Table 1).

Component 2 combines the samples of *A. nodosum* and *P. canaliculata* separating them from the other algae. The most significant constituents of this component are amino acids including both proteinogenic and non-proteinogenic ones (proline, threonine, serine, *O*-acetylserine, pipecolic acid, etc.) and organic acids of the TCA cycle (succinic, fumaric, citric, etc.). Among the other meaningful compounds contributing to component 2 are γ- and δ-tocopherols, sucrose, palmitelaidic acid, phloroglucinic acid derivative, and tetrahydroxybenzene. *A. nodosum* and *P. canaliculata* samples featured rather high relative amounts of tricarboxylic acids of the TCA cycle, tocopherols, pipecolic acid, and tetrahydroxybenzene. The extracts of *S. muticum* and *Fucus* spp. contained relatively more amino acids, palmitelaidic acid, sucrose, and dicarboxylic acids of the TCA cycle (Figure 2, Table 1).

### 2.3. Comparison of Metabolite Profiles of Four Fucus Species

**Figure 3:**
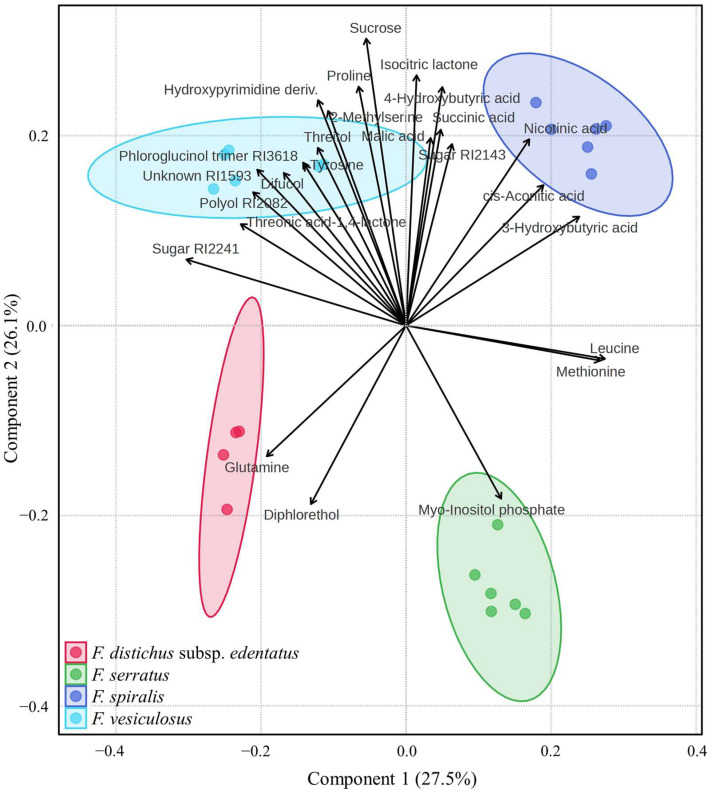
Biplot presenting the sample scores and compounds with the highest loadings for the first two components derived from PLS-DA of the relative metabolite concentrations in the thalli of four species of the genus *Fucus*.

The biplot of PLS-DA accomplished for the data on all studied species representing four genera of the order Fucales showed that samples of *Fucus* spp. tend to form a distinct cluster that is clearly separated from samples of *A. nodosum*, *P. canaliculata*, and *S. muticum* (Figure 2). To analyze the specific biochemical features of different *Fucus* species deeper, another PLS-DA included only the samples of *F. vesiculosus*, *F. serratus*, *F. spiralis*, and *F. distichus* subsp. *edentatus*. The first three components of this PLS-DA covered 72% of the total variance (Figure S2). The biplot presenting the first two components explaining 53.6% of the total variance is shown in Figure 3.

The first component combines samples of *F. vesiculosus* and *F. distichus*, separating them from those of the other two species. Among the compounds showing the highest loadings for this component, the dominant ones are amino acids (leucine, methionine, threonine, etc.), carbohydrates (threonic acid-1,4-lactone, mannitol), phenolic compounds (difucol, phloroglucinol trimer RI 3618, phloroglucinic acid derivative) and, to a lesser extent, fatty acids (palmitelaidic, eicosenoic, eicosapentaenoic acids). Extracts of *F. vesiculosus* and *F. distichus* were relatively rich in carbohydrates and phloroglucinol oligomers and derivatives, whereas the samples of *F. serratus* and *F. spiralis* had relatively high amino acid and fatty acid content (Figure 3, Table 2).

Component 2 reveals common features in the relative biochemical composition of *F. vesiculosus* and *F. spiralis*, contrasting these species with *F. serratus* and, to a lesser extent, *F. distichus*. The top 25 compounds contributing to component 2 include many organic acids (succinic, malic, glyceric, 4-hydroxybutyric acids, etc.) and carbohydrates (sucrose, fructose, glycerol, etc.), as well as four amino acids (proline, glutamine, alanine, 2-methylserine), two phenolic compounds (diphlorethol and homogentisic acid), cholesterol, and linoleic acid. *F. vesiculosus* and *F. spiralis* tend to have a higher relative abundance of carbohydrates (in particular, sucrose) and organic acids, while the samples of *F. serratus* contain relatively high amounts of diphlorethol, glutamine, alanine, *myo*-inositol-phosphate, cholesterol, linoleic, and homogentisic acids (Table 2).

### 2.4. Low-Molecular-Weight Metabolites Particularly Abundant in the Thalli of Certain Fucalean Algae

Each of the studied species of brown algae demonstrated its own unique ‘metabolite signature’ with a high relative abundance of low-molecular-weight compounds of different chemical natures; in particular, carbohydrates and phenolic compounds (Figures S3 and S4). To identify such species-specific sets of relatively abundant marker metabolites, we used the Volcano plot algorithm comparing each species (or genus for *Fucus*) with all others, taken as a group. Table 3 presents a list of compounds having a high relative intensity in one particular species or genus compared to their average levels in the other algae. We considered only differences with a fold change (FC) > 5.

**Figure 4:**
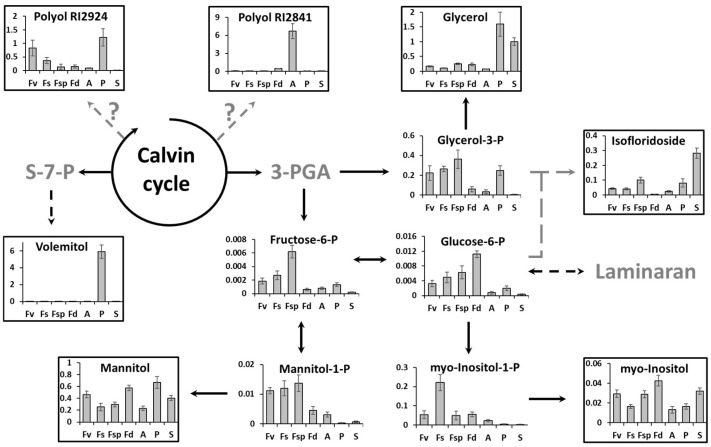
Scheme of the central carbon metabolism and polyol biosynthetic pathways in fucalean algae. Direct reactions are presented as straight lines and reactions involving several steps are presented as dashed lines. Relative metabolite content is given in arbitrary units which are normalized peak areas of extracted ion chromatograms. Metabolites which were not determined, and unknown or putative pathways are labeled in gray. Fv—*Fucus vesiculosus*, Fs—*F. serratus*, Fsp—*F. spiralis*, Fd—*F. distichus* subsp. *edentatus*, A—*Ascophyllum nodosum*, P—*Pelvetia canaliculata*, S—*Sargassum muticum*, S-7-P—sedoheptulose-7-phosphate, 3-PGA—3-phosphoglyceric aldehyde. Bars represent the means ± SD.

As can be seen from Table 3, each algal species contains some metabolites to relative abundances of at least five times higher compared to the corresponding values in other fucoids, and in six out of seven species at least one compound shows more than a tenfold predominance in content. Analysis of this data shows that carbohydrates not only dominate in the cells of fucalean algae but also contribute the most to the biochemical diversity of these organisms. Indeed, various carbohydrates (mono-, di- and trisaccharides, polyols, sugar acids and sugar phosphates) account for ~40% of the compounds listed in Table 3. Some carbohydrates, mostly polyols, could only be found in one particular algal species (e.g., volemitol and polyol RI 2141 in *P. canaliculata*, polyol RI 2096 in *S. muticum*, and polyol RI 3547 in *A. nodosum*), whereas in other studied algae, these analytes were below the limit of detection (Figure 4 and Figure S5).

Other compound groups whose representatives exhibited a high relative abundance specifically in the cells of certain algal species are organic acids (including most of the TCA cycle acids), amino acids (both proteinogenic, such as asparagine and glutamine, and non-proteinogenic, such as γ-aminobutyric and pipecolic acids), and phenolic compounds (mainly phloroglucinol derivatives, low-molecular-weight phlorotannins). In contrast, metabolites associated with lipid exchange showed much more uniform relative content in the thalli of the studied fucoid algae. The only exception here was a high value for palmitelaidic acid in *S. muticum*. Other metabolites demonstrating high species-specific relative intensity were γ-tocopherol, squalene, two nucleosides (adenosine and cytosine), phytol, urea, etc. (Table 3).

## 3. Discussion

### 3.1. Comparison of the General Metabolite Patterns of Fucalean Algae

Our analysis of the GC-MS data allowed the revealing of both specific biochemical characteristics of seven algal species and common features of fucalean metabolome. In general, there are two main reasons for the biochemical similarity between different organisms: their phylogenetic closeness, implying that they share a recent common ancestor, and similar ecological requirements. To date, the phylogenetic relationships within the Fucales are well characterized [25,26,27]. The order currently contains nine families (AlgaeBase, https://www.algaebase.org; accessed on 27 April 2026). The family Sargassaceae, together with Cystoseiraceae, forms a relatively compact monophyletic clade, sister to a larger clade containing the other seven families, including Fucaceae [26]. Thus, among the species used in this study, the biggest phylogenetic distance is between *S. muticum* (Sargassaceae) and all the other algae, belonging to Fucaceae. However, according to PLS-DA, *S. muticum* samples were not clear outliers (Figure 2), indicating that the low-molecular-weight metabolite profile of *S. muticum* has considerable similarity with the metabolomes of the representatives of Fucaceae. In fact, biochemical differences of approximately the same order as those between *S. muticum* and the other algae were observed between the three genera of Fucaceae (Figure 2). We may suggest two explanations for this result. First, despite considerable phylogenetic distance, metabolomes of Fucaceae and Sargassaceae may share enough similar features, common for the whole order Fucales (e.g., relatively high content of carbohydrates and phenolic metabolites). This may also reflect the fact that the GC-MS profiling method detects a high number of primary metabolites in general. The second possible explanation is more intriguing as it considers the specific ecology of *S. muticum*. In contrast to *Fucus*, *A. nodosum*, and *P. canaliculata* that had colonized North Atlantic long ago, *S. muticum* is a non-indigenous species, a newcomer whose established populations were first found in Europe (Isle of Wight, UK) only in 1973 [28]. Nowadays, *S. muticum* occurs on most European Atlantic coasts, and it is still rapidly spreading, making it recognized as one of the most successful invasive species [29]. Such a success in colonization of new vast habitats implies great biochemical plasticity and adaptability of this alga. Thus, we speculate that the acquisition of certain biochemical traits typical for the thriving native species (representatives of Fucaceae) may reflect the biochemical adaptation of *S. muticum* to the North Atlantic habitats. This agrees with the results of Schwartz et al. [30], who compared the biochemical composition of *S. muticum* from Japanese (native) and European populations, showing significant differences for several parameters. In particular, the phlorotannin content in the thalli of *S. muticum* from the North Sea was three times lower than in the native range and close to the values obtained for the North Sea *F. vesiculosus* examined in the same study [30]. It is well known that invasive species often differ in many aspects between their native and introduced ranges; however, most of such data relates to the morphology and ecophysiology of these organisms (e.g., [29,31]), while biochemical changes are still understudied. Certainly, this question needs further research, and comprehensive complementary analyses, such as transcriptomics, enzymatic assays, as well as physiological experiments are necessary to experimentally validate the hypotheses based on metabolomics data.

As for the phylogenetic relationships within the family Fucaceae, this group is quite complex. It most likely originated in the Pacific Ocean during the mid-to-late Miocene, and the ancestors of the Atlantic genera of these algae entered the Atlantic Ocean during the last opening of the Bering Strait [25]. The colonization of the Atlantic involved four independent trans-Arctic crossings, accompanied by the split of the phylogenetic lineages, which finally led to the emergence of *P. canaliculata*, *A. nodosum*, the genus *Fucus*, and *F. distichus sensu lato* [27]. According to phylogenetic trees of Fucaceae based on molecular data, the biggest distance separates *A. nodosum* and *Fucus* spp., while *P. canaliculata* is closer to *Fucus*. As for the different species of the genus *Fucus*, they form two phylogenetic lineages: the first clade contains *F. serratus* and *F. distichus* (cold-water and relatively stress-sensitive algae), and the second clade unites six species, including *F. vesiculosus* and *F. spiralis* (algae with a more southerly range and higher stress tolerance) [25,27,32]. Furthermore, again, beyond the distinct clustering of all *Fucus* species sharing a number of specific biochemical features (Figure 2 and Figure S1, Table 3), metabolomics results do not clearly reflect phylogenetic relationships within the Fucaceae, showing a more pronounced difference between *Fucus* and *P. canaliculata* than between *Fucus* and *A. nodosum* (Figure 2). Moreover, a detailed comparison of four *Fucus* species revealed that the most biochemically similar fucoids in this study are *F. vesiculosus* and *F. distichus* subsp. *edentatus* (Figure 3), with the species belonging to different lineages, but occupying close positions on the seashore. *F. distichus* subsp. *edentatus* typically inhabits shallow subtidal–low-intertidal zones, and *F. vesiculosus* is a low- to mid-intertidal species (Table 4), so their habitats partially overlap and, in locations where both algae occur (e.g., in the White Sea), they frequently grow together in the low-intertidal zone of the shore. In contrast, phylogenetically closer *F. vesiculosus* and *F. spiralis* do not share common habitats, as *F. spiralis* is a high-intertidal species (Table 4). Thus, we suggest that ecological factors mainly determine the specific biochemical features of these algae. In this case, the pronounced difference between the metabolome of *P. canaliculata* and those of the other two genera of Fucaceae does not seem counterintuitive, considering the unique environment of *P. canaliculata*. This miniature fucoid forms a narrow belt on the wave-exposed rocks at the uppermost boundary of the intertidal zone, where it constantly encounters extreme environmental conditions. In fact, *P. canaliculata* spends most of its life exposed to air, as it is submerged for no more than 8 h per day during spring tides and may remain dry for several days during neap tides [21]. Undoubtedly, adaptation to such a specific habitat required profound biochemical changes and led to the formation of a unique metabolite profile (Figure 2, Table 3). Common features in metabolomes of *P. canaliculata* and *A. nodosum* revealed by PLS-DA (Figure 2 and Figure S1) may also derive from the ecophysiological specificity of these organisms. Both algae form permanent intimate associations (mycophycobiosis) with endophytic ascomycete fungus *Stigmidium ascophylli*, giving them some lichen features [33,34]. Thus, they might develop similar biochemical adaptations, such as relatively high content of low-molecular-weight carbohydrates and tricarboxylic acids, to interact with their fungal symbiont (Figure 1, Table 1).

The main source for ambiguities in the comparison of phylogenetic and biochemical data on four *Fucus* species is most likely the unusually complicated phylogeny of *F. distichus*, as this entity currently includes many subspecies and forms considerably varying in their morphology and ecological preferences [27,35]. In particular, *F. distichus* subsp. *edentatus* (former *F. edentatus*) used in this study differs greatly from typical *F. distichus* in both size of the thallus and the position on the shoreline. In contrast to high-intertidal *F. distichus* inhabiting shallow rock pools and having relatively small thalli, *F. distichus* subsp. *edentatus* is a large seaweed (similar to *F. vesiculosus* in size) growing in the subtidal–low-intertidal shore zones (Table 4). The biochemistry of *F. distichus* subsp. *edentatus* is currently very poorly studied, though our previous research showed that at least some parameters differ considerably between different members of the *F. distichus* complex [36].

To conclude this chapter, it should be emphasized here that our study is based entirely on GC-MS metabolomics and therefore has certain associated limitations. Involving additional approaches relying both on other “omics”-techniques and ecophysiological experiments might be needed to obtain more complete and meaningful data on general and specific biochemical traits of fucalean algae. Moreover, algal samples in this study were collected from two different geographical locations (Table 4), and although data analysis (Figure 2, Figure 3 and Figures S1–S4) did not reveal any sample clustering which might be attributed to this factor, some site-specific environmental parameters may also have contributed to the observed differences between algal species. A logical continuation and development of this research would be larger-scale long-term studies, including samples collected in different geographic regions and during different seasons.

### 3.2. Polyols of Fucalean Algae

The most notable common feature of all tested algae is the high contribution of carbohydrates to their low-molecular-weight metabolomes (Figure 1). Analysis of the molecular profiles of these compounds showed that polyols are the preferred type of carbohydrates accumulating in algal cells. Generally, this result corresponds well to the literature data on several fucalean, as well as ectocarpalean and laminarialean algae, where mannitol was reported as the major carbohydrate with contents, in most cases, ranging from 103 to 347 μmol g^−1^ of dry weight (DW), while the amount of dominant sugar, glucose, was 2–3 orders of magnitude lower [14,21,37,38]. Thus, mannitol seems like a ubiquitously dominant polyol in brown algae, particularly in Fucales. However, our results suggest that several other mono-, di- and trisaccharide-based alcohols can also accumulate in cells of fucalean algae in considerable concentrations, sometimes comparable to mannitol. These include, first, volemitol (a metabolite specific to *P. canaliculata* [39]), as well as glycerol, isofloridoside, inositol, and a number of polyols whose structure could not be identified unambiguously (Table S1).

Sugar alcohols and their derivatives are formed in cells of brown algae as major stable products of photosynthesis ([40]; Figure 4). As such, these compounds successfully combine several valuable features. Compared to other low-molecular-weight photoassimilates, polyols are highly reduced substances with relatively low reactivity, allowing them to accumulate to high concentrations without interfering with intracellular biochemical processes. Therefore, polyols comprise an efficient sink for photosynthetically fixed carbon and a capacious pool of energy-rich storage compounds. In addition, these metabolites have considerable osmoprotective properties and can serve as compatible solutes for osmotic stress mitigation [41]. Numerous research on vascular plants showed that enhanced production of sugar alcohols improves tolerance to stress conditions and unstable resource availability [42]. It has also been suggested that organisms that preferentially invest photosynthates into relatively expensive and metabolically inert products, such as polyols, might employ a specific growth strategy resulting in the limitation of growth rate and productivity [41]. All this is in good agreement with the biology of intertidal fucalean algae, perennial organisms characterized by relatively low growth rates but extremely high stress resistance which ensures their survival and successful reproduction in harsh environments, where they are systematically subjected to changes in humidity, light intensity, temperature, salinity, nutrient availability, etc. [43].

Interestingly, comparison of the metabolite profiles of fucalean algae showed that representatives of the genus *Fucus* tend to invest photoassimilates almost exclusively in mannitol, whereas *A. nodosum*, *P. canaliculata* and *S. muticum* seem to have more complicated machinery of carbon partitioning and a more diversified spectrum of polyols serving as stable photosynthetic products (Figure 4). Thus, the total intensity of the carbohydrate-related signals in TIC chromatograms strongly correlated (*r* = 0.97) with relative content of mannitol for four *Fucus* species, but for the whole list, including seven fucalean species, the correlation was considerably lower (*r* = 0.67) (compare data from Figure 1 and Figure 4).

Information on details of primary metabolism of brown algae is still rather fragmentary. Moreover, except for few targeted pathways, metabolism of polyols remains relatively poorly characterized even in vascular plants [41]. Most comprehensive studies on polyol biosynthesis in Phaeophyceae relate to mannitol: this metabolite is synthesized from triosephosphates derived from the Calvin cycle via fructose-6-phosphate and mannitol-1-phosphate (Figure 4; [40]). The other possible ways of using triosephosphates for biosynthesis of sugar alcohols lead to the formation of inositol, as well as glycerol (specifically accumulated in *P. canaliculata* and *S. muticum*) and its derivatives, such as glycerol-glycosides (Figure 4). One of the glycerol-derived compounds specifically accumulated in *S. muticum* deserves particular attention. In our study, this analyte was annotated as isofloridoside; spectral information supporting this tentative identification is provided in Figure S6. Since spectral library matching alone does not provide sufficient information to clearly differentiate the two isomers, isofloridoside and floridoside, our annotation was based on differences in the RI values of these compounds. Both isomers are included in our in-house library with accurate RI information; in particular, the library RI value for floridoside is 2255.2, while RI value for isofloridoside is 2310.9, which is close to the RI of the metabolite in question (2327.4, Table S1). Thus, annotating this metabolite as isofloridoside we should emphasize that this is a putative identification based on RI and spectral library matching and that it needs further confirmation.

Together with its isomer floridoside, isofloridoside is best known as a typical metabolite of Rhodophyta. In red algae, these glycerol-galactosides are dominant low-molecular-weight carbohydrates, the main products of photosynthesis, soluble storage compounds, and osmolytes, i.e., metabolites having the same functional roles as mannitol in brown algae [44,45]. Besides red algae, floridoside and/or isofloridoside were also found in chrysophytes, cryptophytes, dinoflagellates, and cyanobacteria [46,47,48]; but, to our knowledge, this study is the first finding of (iso)floridoside in representatives of Phaeophyceae. Apparently, synthesis of these galactosides is an evolutionary conserved metabolic pathway. In red algae, (iso)floridoside is produced by the condensation of glycerol-3-phosphate and UDP-galactose, followed by a dephosphorylation reaction [47]. Glucose-1-phosphate derived from either the Calvin cycle or degradation of floridean starch serves as a precursor to UDP-galactose [49]. We hypothesize that in brown algae, the breakdown of storage polysaccharide laminaran may also be used for this purpose (Figure 4).

Another dominant polyol, which in our study was second only to mannitol in accumulation, is volemitol, a seven-carbon compound detected exclusively in the samples of *P. canaliculata*. This is entirely consistent with published data indicating that volemitol is a specific metabolite of *P. canaliculata* [39,50]. Similar to other major sugar alcohols of brown algae, biosynthesis of volemitol is tightly linked to central carbon metabolism. This heptitol is formed in *P. canaliculata* cells from sedoheptulose-7-phosphate via volemitol-1-phosphate ([50]; Figure 4). The other two relatively abundant carbohydrates contributing considerably to biochemical diversity of the studied algae are polyols RI 2841 and RI 2924 (Figure 4, Table S1). Based on their MS-spectra and RI values, we conclude that these are disaccharide-based polyols. We hypothesize that the yet unknown polyol compounds of this type, together with some larger, trisaccharide-based molecules (e.g., polyols RI 3391 and RI 3547) may be intermediates of the metabolism of laminaran, a polydisperse polymer based on β-1,3-glucose and containing up to 3.7% mannitol (subject to future confirmation) [51]. The biosynthetic pathway of laminaran in brown algal cells is still not fully elucidated; however, both mannitol-glucopyranoside and mannitol-diglucopyranoside have been found in *Fucus* [51,52].

In general, the proportion of carbohydrates (mainly represented by polyols) in the profiles of low-molecular-weight metabolites of six species of Fucaceae is in good agreement with their ecological features, such as vertical distribution on the seashore; the lowest relative content of carbohydrates was shown in the subtidal *F. serratus*, and the highest in the high-intertidal *P. canaliculata* (Table 4, Figure 1). These data support the hypothesis that polyols play a key role in stress tolerance and particularly desiccation.

The high capacity for synthesizing and accumulating polyols is a valuable biochemical feature making fucalean algae demanded from the practical perspective. The production of mannitol from seaweed has received considerable attention in recent years [53]. Sugar alcohols, and in particular, mannitol, are widely utilized in various fields of industry such as food and pharmaceutical production [53,54,55]. These compounds are popular sweeteners, flavor enhancers, stabilizers, and moisturizing agents [54]. Mannitol is a component of medications used for treatment of inflammation, diabetes, neural and renal insufficiency, ocular and intracranial hypertension, etc. [53,55]. Moreover, because of its high heat value property, mannitol is regarded as an effective source of biofuels, particularly bioethanol [53,56].

### 3.3. Phenolic Metabolites of Fucalean Algae

The general presence of phenolic metabolites in brown algae, particularly in the representatives of the order Fucales, is studied abundantly with a focus on analytical methods (reviewed by [57]), physiological and ecological implications [58,59], and practical relevance of these compounds (reviewed by [60]). The dominant phenolics occurring in brown algae are diverse phloroglucinol-based polymers named phlorotannins. These molecules play a structural role when incorporated into the algal cell walls but can also serve various important purposes in seaweed chemical defense [59,61]. Consequently, their content varies with water salinity, nutrient availability, plant habitat, size and developmental stage, season, grazing intensity, and other conditions [59]. It was also shown that environmental stressors, such as light, temperature, nutrient availability, and grazing can contribute to the production of phlorotannins with greater structural diversity [62,63]. Thus, Arnold and Targett [64] reported a significant turnover of phlorotannins in algal cells, both in laboratory and in natural conditions, but varying in different brown algal species, which might indicate a difference in the use of these compounds. In view of these diverse functions and considerable biological activity of these compounds, research into the structure–activity relationship (SAR) of specific phlorotannins has intensified in recent years, and interest in comprehensively understanding the connection between different molecular species has grown [6,65].

**Figure 5:**
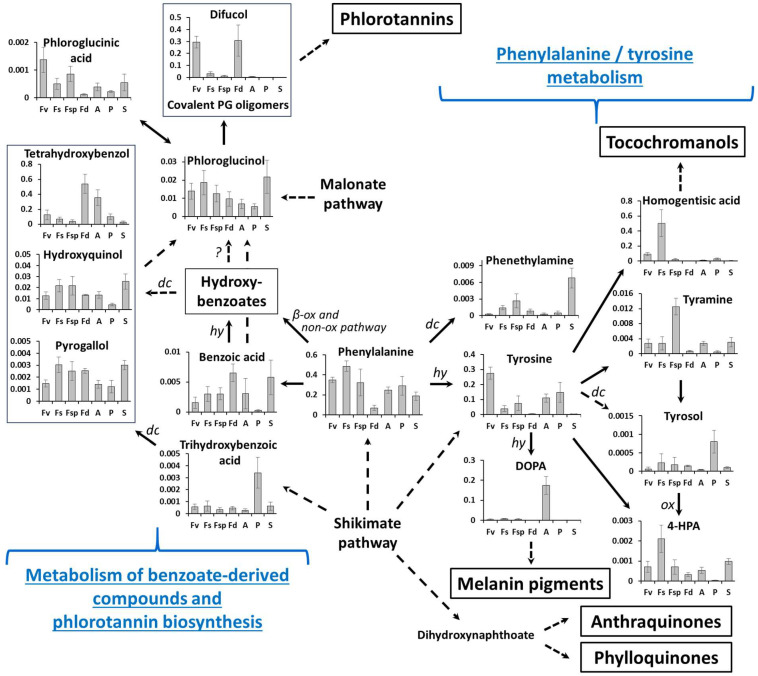
The scheme of the metabolic pathways of phenolic compounds in fucalean algae. Direct reactions are presented as straight lines and reactions involving several steps are presented as dashed lines. Relative signal intensities are given in arbitrary units, which are normalized peak areas of extracted ion chromatograms. Fv—*Fucus vesiculosus*, Fs—*F. serratus*, Fsp—*F. spiralis*, Fd—*F. distichus* subsp. *edentatus*, A—*Ascophyllum nodosum*, P—*Pelvetia canaliculata*, S—*Sargassum muticum*, DOPA—dihydroxyphenylalanine, 4-HPA—4-hydroxyphenylacetic acid, PG—phloroglucinol, *dc*—decarboxylation, *hy*—hydroxylation, *ox*—oxidation. Bars represent the means ± SD. The confidence level of the tentative identifications for the metabolites included in this scheme is given in Table S1.

Across the whole dataset obtained in our study (Table S1), about 50 compounds were regarded as being of phenolic origin and several other metabolites (e.g., benzoic acid and phenylalanine) are closely associated with the metabolism of phenolic compounds. Overall, metabolites of phenolic or aromatic natures account for ~20% of the annotated metabolome of analyzed fucalean algae. About two thirds of these compounds (including analytes with tentative annotation) may be involved in the formation of phlorotannins. The biosynthesis of the phlorotannin monomer, phloroglucinol, is not fully elucidated yet, but is suggested to occur via condensation of acetate and malonate units, with 1,3,5-cyclohexatrione as intermediate to phloroglucinol [66,67]. Alternative hypotheses are the formation of the triol by the shikimate (SA) or phenylpropanoid pathways ([67]; Figure 5).

Interestingly, apart from the suggested starting products of phlorotannin polymerization from phloroglucinol, namely the phloroglucinol dimers (difucol and diphlorethol), the tentatively identified hydroxylated dimers, as well as two trimers, we also annotated alternative phenolic monomers such as a tetrahydroxybenzene, the two other trihydroxybenzenes, hydroxyquinol, and pyrogallol, as well as phloroglucinic acid and a tentative derivative (Table S1). Some of these compounds, such as pyrogallol, were already previously reported in brown algae of the orders Fucales and Laminariales [68,69]. Moreover, a phlorotannin oligomer having a pyrogallol moiety in its structure (pyrogallol-phloroglucinol-6,6-bieckol) was isolated from the laminarialean alga *Ecklonia cava* [70]. The fact that SA-derived gallic acid is usually considered to be the precursor of pyrogallol [71] might imply in analogy that decarboxylation of phloroglucinic acid could open an alternative path to ensure accessibility of the central phlorotannin monomer phloroglucinol (Figure 5). Like phloroglucinol, the hydroxylated monomers pyrogallol and hydroxyquinol are known to be rather susceptible to strong non-covalent interactions (e.g., multiple hydrogen bonding, and electrostatic and cation–p interactions) as well as covalent interactions (e.g., Michael addition/Schiff-base reaction, radical coupling reaction, and dynamic coordination interactions with metal ions) giving rise to their involvement in formation of diverse compounds [72,73]. For instance, an alternative biosynthetic pathway of phloroglucinol was shown from hydroxyquinol and pyrogallol, with tetrahydroxybenzene as cosubstrate in the anaerobic bacteria *Pelobacter acidigallici* and *P. massiliensis* [74,75].

The occurrence and biosynthesis of phloroglucinic acid, however, have not been subject to extensive research yet, which is usually overshadowed by research on the much more targeted phloroglucinol. Nevertheless, its presence has been reported in many other works, along with other phenolic (i.e., homogentisic) and (hydroxy) benzoic acids (e.g., [14,24,68]). Phenolic and benzoic acids are also described to derive from the shikimate and the phenylpropanoid pathways (which again may suggest a regulated biosynthetic connection between phloroglucinol and its acid) [76]. For example, homogentisic acid is an intermediate in the catabolism of aromatic amino acids such as SA-derived tyrosine and a precursor for the synthesis of tocopherols [77]; benzoic acid is produced from phenylalanine-derived cinnamic acid [78]. Interestingly, however, apart from targeted biosynthesis and, similar to pyrogallol and gallic acid, it was also described that phloroglucinic acid can form spontaneously from phloroglucinol in aqueous, carbonate-containing solution [79].

Given the importance of phenolic compounds among phytochemicals, we had a deeper look at whether we could observe a similar pattern of certain phenolic groups across algal species pointing to physiological relationships. A PLS-DA and a hierarchical cluster analysis of 37 phenolic compounds (Figures S4 and S7) suggested that *P. canaliculata* had the most distinct pattern among the analyzed algae, which is consistent with the analysis of the entire dataset (Figure 2). However, as illustrated in the heatmap (Figure S4), all studied algal species presented rather specific phenolic profiles. Notably, many phlorotannin-related analytes (phloroglucinol dimers and trimers, putative benzene-triol derivatives, and triolbenzoate glycosides, etc.) were clearly correlated with each other, showing the highest relative abundance in *F. vesiculosus* and the lowest in *P. canaliculata*. Although some of these analytes were only tentatively identified in this study, such clear clustering supports our annotation. The second distinct cluster combined the three triols (phloroglucinol, pyrogallol, and hydroxyquinol), indicating a close metabolic relationship between them in the algal cell. Then, another cluster included two SA-derived compounds, phenylalanine and homogentisate, with the highest relative abundance in *F. serratus* (Figure S4). Apparently, this rich pattern reflects the general complexity of metabolism of phenolic compounds in fucalean algae with multiple “molecular hubs” channeling the biochemical resources and regulating the whole process.

Based on the phenolic profiles of fucalean algae and information on possible routes of phlorotannin biosynthesis (Figure 5), we may suggest that different pathways may contribute unevenly to phloroglucinol/phlorotannin formation in different algal species. Thus, for *F. vesiculosus*, which had a relatively low content of various phenolic monomers but was rich in oligomers, the *malonate pathway* may play a key role. In contrast, *S. muticum* and, to a lesser extent, *F. serratus*, had a high relative abundance of the trihydroxylated phenolic monomers (as did *F. distichus*, with respect to benzoic acid and tetrahydroxybenzene and *P. canaliculata*, with respect to hydroxyquinolinic acid) which may indicate a rather active branch of the *shikimate pathway* associated with the metabolism of benzoate-derived compounds. Unfortunately, the basic components of these two metabolic pathways, such as malonyl-CoA as well as shikimate or prephenate, were not detected using the method employed. For *F. serratus*, which demonstrated relatively high levels of phenylalanine and phloroglucinol but low abundance of benzoate, the ß-oxidative and non-oxidative pathway to phloroglucinol/phlorotannin formation may also be suggested (Figure 5).

Phenolic compounds associated with the metabolism of phenylalanine and tyrosine also showed species-specific molecular profiles in our study (Figure 5 and Figure S4). Notably, *A. nodosum* had the highest content of 3,4-dihydroxyphenylalanine (DOPA), accompanied by relatively low levels of other tyrosine derivatives (tyrosol, homogentisic acid, etc.), suggesting an active *phe/tyr pathway* focused on DOPA production. Interestingly, *A. nodosum* is known for its high phlorotannin content [80], which again suggests that the malonate pathway may play a more important role in the formation of phlorotannins in this organism as well. In *F. serratus*, instead of DOPA, phenolic acids (homogentisic and 4-hydroxyphenylacetic, 4-HPA) were most abundant, suggesting other consumption routes for the SA-derived amino acids. DOPA and 4-HPA produced from tyrosine can be precursors to catecholamines or melanin-like pigments, e.g., via tyrosinase-mediated oxidation; in some photosynthetic organisms, DOPA-derived pathways contribute to phenolic pigments and UV/oxidative stress protection [81,82]. As for homogentisate, this compound is a metabolic precursor of tocochromanols [77]. Finally, *F. spiralis* had the highest abundance of tyramine, as well as dihydroxynaphthoic acid (Figure 5 and Figure S4), which is a precursor of phylloquinones and anthraquinones [83].

In summary, though the components of the main pathways were detected to be present across all investigated species, it seems that each species relies on a specific profile of the considered aromatic compounds: *A. nodosum* favored precursors of melanin-like pigments, *F. spiralis* those of anthra- and phylloquinones, *F. serratus*—those of tocochromanols, and *F. vesiculosus*, *F. distichus*, *S. muticum*, and *P. canaliculata* the ones of phlorotannins. Regarding *P. canaliculata*, however, the intermediate relative abundances in most phenolic monomers contrasting the low relative abundance of phloroglucinol dimers and trimers, are in good agreement with earlier reports that this species is particularly rich in phlorotannins of higher degrees of polymerization in contrast to *F. vesiculosus*, where low-molecular-weight phlorotannins predominate [24].

Phlorotannins, melanin pigments, tocochromanols, and naphthoquinones, including anthraquinones, are all valuable phytochemicals considered to protect the organism against oxidative stress [84,85,86,87]. Furthermore, plant and algal phenolic compounds represent a very important source of nutrition and medical applications for humans, where they can be of great benefit due to their antioxidant, anti-inflammatory, and antimicrobial effects. In particular, phlorotannins have been extensively studied as promising substances, not only mitigating oxidative stress but also interacting with key signaling pathways involved in inflammation and acute or chronic diseases (e.g., [88,89]), though the clinical implementation of such applications would still require rigorous pharmacokinetic, safety, and efficacy research [90]. Other phenols considered in our study are also promising lead compounds for drug development or as complementary nutraceuticals [85,91].

### 3.4. Organic Acids of the TCA Cycle

**Figure 6:**
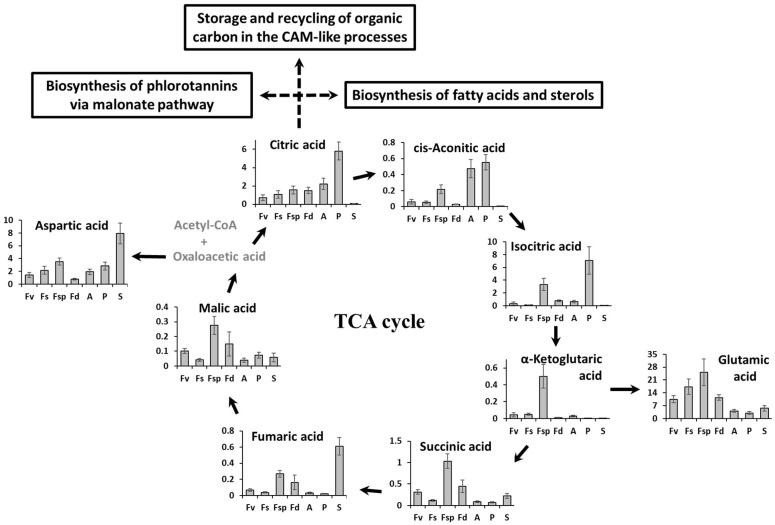
Specific accumulation and presumable consumption pathways of TCA cycle metabolites in the thalli of seven species of fucalean algae. Direct reactions are presented as straight lines and reactions involving several steps are presented as dashed lines. Relative metabolite content is given in arbitrary units which are normalized peak areas of extracted ion chromatograms. Metabolites which were not determined are labeled in gray. Fv—*Fucus vesiculosus*, Fs—*F. serratus*, Fsp—*F. spiralis*, Fd—*F. distichus* subsp. *edentatus*, A—*Ascophyllum nodosum*, P—*Pelvetia canaliculata*, S—*Sargassum muticum*. Bars represent the means ± SD.

According to our analysis, organic acids of the TCA cycle comprise one more group of metabolites that contributes considerably to the biochemical diversity of fucalean algae (Figure 1 and Figure 2, Table 1, Table 2 and Table 3). Moreover, one of these compounds, citric acid, was among the dominant metabolites of the studied seaweed. This result is in agreement with the literature data: relatively high content of tricarboxylic acids, and in particular citrate (up to 32 μmol g^−1^ DW, for *P. canaliculata*), seems a general biochemical feature of fucalean algae [14,21] and, possibly, all brown seaweed, as this was also shown for representatives of Ectocarpales and Laminariales [37,38,92]. At the same time, content of dicarboxylic acids of the TCA cycle in the same algae may be 3–4 orders of magnitude lower [14,21]. Such disproportion suggests some specific functions for citric acid in brown algal cells, apart from its role in cell respiration. Citrate stands at the crossroads of several metabolic pathways and can easily leave the TCA cycle and mitochondria to provide organic carbon for different cell biosynthetic processes, such as the formation of fatty acids and sterols [93]. What is especially interesting in the context of brown algae, is that citrate may be regarded as a key source of malonyl-CoA for phlorotannin production via the acetate–malonate pathway (Figure 5 and Figure 6). Indeed, cytosolic citric acid may be cleaved by ATP-citrate lyase to produce acetyl-CoA, which in turn is converted to malonyl-CoA by acetyl-CoA carboxylase [93]. This mechanism puts together two known biochemical peculiarities of brown algae: a tendency to accumulate relatively high content of citrate and the constant biosynthesis of great amounts (up to 26% DW, [24]) of phlorotannins. Both features are especially pronounced in the representatives of the order Fucales [6,14,21,24,94].

According to our data, the highest concentration of citrate was found in the thalli of *P. canaliculata* (Table 3; Figure 6). Specific accumulation of this compound in the high-intertidal alga (Table 4) may relate to one more possible metabolic role of citric acid: storage and recycling of organic carbon in processes similar to CAM-photosynthesis, which contribute to maintaining the photosynthetic activity of *P. canaliculata* thalli exposed to air during the low-tide periods. In our previous study [21], we showed that the tidal cycle induces considerable changes in titratable acidity of *P. canaliculata* thalli, and these changes are underpinned by oscillations of citrate concentration. Contribution of citric acid into CAM-photosynthesis was shown for many terrestrial CAM plants also accumulating relatively high amounts of this metabolite (reviewed in: [95]). Thus, we suggest that the specific accumulation of citric acid in *P. canaliculata* may reflect an increased need for this compound caused by the specific ecological preferences of this alga.

Among the other fucalean species considered in this study, *F. spiralis* draws attention, exhibiting the highest contents of several dicarboxylic acids, such as α-ketoglutaric, malic, and succinic ones (Table 3, Figure 6). This may also be explained from the perspective of the biosynthetic roles of the TCA cycle intermediates, since the dicarboxylic acids, α-ketoglutarate, and oxaloacetate (derived from malate) serve as “switch valves” for the efflux of organic carbon from the cycle for amino acid biosynthesis [93]. Accordingly, samples of *F. spiralis* were characterized by relatively high levels of the direct derivatives of the TCA cycle metabolites, namely glutamic and aspartic acids (Figure 6). These results are in good agreement with the data of Mouritsen et al. [96], who compared free amino acid profiles of six representatives of the order Fucales and showed a specific accumulation of glutamate and aspartate in *F. spiralis*. Both metabolites are known as dominant free amino acids in brown algae (e.g., [14,37,96]).

Data on the specific accumulation of different organic acids and metabolically linked amino acids in different fucoids can be useful for the valorization of seaweed biomass and establishment of biorefinery systems focused on the most waste-free exploitation of this resource.

## 4. Materials and Methods

### 4.1. Algal Material Collection

Mature thalli of *Fucus vesiculosus* L., *F. serratus* L., *F. spiralis* L., *Ascophyllum nodosum* (L.) Le Jolis, and *Sargassum muticum* (Yendo) Fensholt were collected at the island of Helgoland (North Sea, Germany). Thalli of *Pelvetia canaliculata* (L.) Dcne & Thur. and *F. distichus* subsp. *edentatus* (Bachelot Pyl.) H.T.Powell were collected in the Keret Archipelago (Kandalaksha Bay, White Sea, Russia). At both sites, sampling was carried out in the beginning of the vegetative season (May for the North Sea and June for the White Sea) in the typical habitats of each species (Table 4) during ebb or low tide, from underwater (for subtidal algae) or shortly after emerging (for intertidal algae). When the algae grew as wide bands on the shore, samples were taken from the middle zone of the corresponding fucoid belt. Algal thalli were delivered to the laboratory in seawater, thoroughly washed and cleaned of epiphytes, and then either subjected to immediate extraction or frozen until further processing.

### 4.2. Metabolite Profiling

Before starting the sample preparation, feasibility and appropriate sample amount were assessed in pilot experiments with ascending amounts of algal material. Fragments of algal thalli (20 mg FW) were poured with cold methanol (−25 °C), quickly ground in a pre-cooled mortar, then sonicated for 10 min (Emag GmbH + Co. KG; Salach, Germany) and left soaking in 1 mL of cold methanol for extraction. Aliquots of 500 μL of methanol extracts were transferred to clean 1.5 mL polypropylene Eppendorf tubes (Eppendorf SE, Hamburg, Germany) and vacuum-dried in the CentriVap vacuum concentrator system (Labconco, Kansas City, MO, USA) for subsequent analysis.

Metabolite profiling analyses were carried out according to Hutschenreuther et al. [97]. Briefly, vacuum-dried extracts were incubated by shaking in methoxyamine hydrochloride (Alfa Aesar by Thermo Fisher Scientific, Kandel, Germany) solution in pyridine (Sigma-Aldrich, Taufkirchen, Germany) and *N*,*O*-bis(trimethylsilyl)-trifluoroacetamide (CS Chromotagraphie Service GmbH, Langerwehe, Germany). After derivatization, samples were transferred to glass vial micro-inserts and subjected to GC-MS analysis on an Agilent 6890 gas chromatograph coupled to an Agilent 5973N quadrupole mass selective detector (Agilent Technologies, Böblingen, Germany) with standard electron impact ionization (70 eV). Separation was accomplished on a DB-5MS Ultra Inert column (Agilent, Waldbronn, Germany; 30 m × 0.25 mm ID and 0.25 µm film) at 0.9 mL/min carrier gas flow (He 5.0 Alphagaz, Air Liquide, Frankfurt, Germany) after splitless injection at 280 °C. Within each sequence, signals of validated retention behavior were used for the calculation of Kovats retention indices. A mix of authentic reference standards, containing 21 amino acids, 23 sugars and polyols, 19 organic acids, phloroglucinol, etc., was co-spiked to confirm the identity and RI of expected compounds. To avoid potential bias when using a single internal standard [97], we based our analysis on normalization using a parameter intrinsic to the chromatogram (see below).

Peak deconvolution was accomplished using AMDIS 2.71. The retention indices were automatically calculated using an AMDIS calibration file containing the batch retention times of each alkane. GMD (Golm metabolome database, GMD_20100614_VAR5_ALK, [98]) and NIST14 (National Institute of Standards and Technology, Gaithersburg, MD, USA) were used for tentative identification of the peaks based on automated and manual spectra comparison. The analytes that could not be attributed to certain compounds were annotated to specific chemical classes by the presence of characteristic fragment ions (*m*/*z* ± 0.5) in their extracted ion mass spectra. The following *m*/*z* values of the fragment ions were used according to Harvey and Vouros [99]: 100 and 174—amino group-containing metabolites; 117—fatty acids; 299, 315, 357 or/and 387—phosphate group-containing compounds; 103, 160, 217, 307/319—C6-monosaccharides; 204 and 319—sugar alcohols and glycosides, respectively, pyranoses, 318 and 319—stereoisomers of inositol, 292, 333 and 319—sugar derived acids, 361, 437 or/and 451—di- and trisaccharides. To identify further phenolic compounds in the metabolic profiles for which no reference spectrum was available in mass spectral libraries, a literature review on the metabolic pathways of phlorotannins and the results of a previous, detailed LC-MS study on these metabolites [24] were utilized. For these compounds, the *m*/*z* values of the expected molecular ion of the corresponding derivative and the M-15 were used, as aromatic substructures stabilize the molecular ion. The assignment of the relevant spectra was then refined through manual spectral interpretation using diagnostic ions for a particular compound group (benzene-triols, triolbenzoates, etc.). All these identifications are tentative assignments and subject to further confirmation in upcoming investigations.

### 4.3. Data Analysis

Experiments were carried out with 4–6 biological replicates (taken from different individuals). Quantitation of metabolites in GC-MS analysis was performed by peak integration of the corresponding extracted ion chromatograms (*m*/*z* ± 0.5) for representative intense signals at specific retention times (RT) using Xcalibur 3.0. Excel 2013 (Microsoft, Redmond, WA, USA) and MetaboAnalyst 6.0 Web application (http://www.metaboanalyst.ca, accessed on 5 June 2026), which were used for data processing and normalization procedures, creation of figures, and heatmap construction. Metabolites of rather low overall abundance needed to be excluded from further processing, which relates to 17 tentative phenolic compounds in the dataset. Metabolomic data processing included removal of features detected in less than 50% of replicates, peak area normalization to the sum of all areas (TIC) within the corresponding chromatogram, generalized logarithm transformation, and range data scaling (mean-centered and divided by the range of each variable). In TIC-based normalization, the relative intensities of the target substances are adding up to 100% without absolute quantification, which does not allow for comparisons of physiological metabolite concentrations, particularly where their metabolite profiles differ significantly. Chromatogram-intrinsic normaliation such as TIC normlization works best when total metabolite abundance is similar across samples, which can become quite a challenge when comparing species with very different metabolic profiles (e.g., *P. canaliculata* vs *F. serratus*). As this could lead to potential distortion of the relative abundances of certain compounds in worst cases [97], we tested other normalization strategies such as median-normalization or other statistical methods such as classical principal component analysis to validate our results, where we obtained similar results.

All values are expressed as means and standard deviations.

## 5. Conclusions

We analyzed metabolite data from seven species of Fucales within the context of physiological and ecological characteristics of the different algae, thus revealing the taxon-specific biochemical signatures and metabolite patterns contributing to seaweed adaptation to various habitats and environmental conditions. Analyzing three important groups of metabolites representing polyols, phenolic compounds, and TCA cycle organic acids in more detail, we were able to considerably extend the scope of mass spectral library annotations by manual spectral interpretation, such as for various low-molecular-weight phenolics contributing to ~20% of the total annotated metabolome. Thereby, we showed how traditional GC-MS-based metabolomics can contribute to boost structural elucidation in exploring the diversity of valuable compounds of seaweed origin and to reveal the algal species synthesizing and specifically accumulating such substances.

We found similar metabolic characteristics not only among representatives of the same genus (*Fucus* spp.), but also within higher taxonomic groups. At the same time, some species were characterized by distinctly high abundances of certain metabolites. Interestingly, the latter phenomenon was not only related to secondary metabolites such as, for instance, a hydroxyquinolinic acid (*P. canaliculata*) and homogentisate (*F. serratus*), but also to primary metabolites like those of the TCA cycle: citrate/isocitrate in *P. canaliculata*, α-ketoglutarate in *F. spiralis*, and fumarate in *S. muticum*. In conclusion, we suggest that these distinctive metabolic characteristics are not only related to the phylogenetic origin of species, but also partly reflect physiological features such as size, lifespan, and ecological preferences, respectively, as well as specific requirements in current habitats. While it may complicate the prediction of the presence of valuable natural compounds in algae of the same species, this fact makes metabolite profiling an indispensable tool for researching and assessing the benefits of a particular algal species for human use.

## Figures and Tables

**Table 1:** Tentatively identified compounds with the highest loadings in the first two components derived from PLS-DA of metabolite profiles of seven species of Fucales. The vertical order in the table is according to the absolute values of the loadings—for each component, the compound with the highest value is in the first line. Signs in the brackets mean positive or negative values.

Component 1	Component 2
Carbohydrate RI 2028 (−)	Pyrrole-2-carboxylic acid (+)
Erythronic acid (+)	Proline (+)
Polyol RI 2834 (−)	Threonine (+)
Disaccharide RI 2879 (−)	Serine (+)
Asparagine (−)	Succinic acid (+)
Glycerol (+)	Lyxonic acid (−)
Mannitol (+)	O-acetylserine (+)
Trehalose (+)	Phloroglucinic acid derivative (+)
Glucose (−)	Asparagine (+)
Pyroglutamic acid (−)	S-methylcysteine (+)
Volemitol (+)	Palmitelaidic acid (+)
Oleic acid (−)	Valine (+)
Dihydroxyphenylalanine (−)	2-Methylserine (+)
α-Tocopherol derivative (−)	Fumaric acid (+)
Isofloridoside (+)	Urea (−)
α-Ketoglutaric acid (−)	Arabitol (−)
Citramalic acid (+)	β-Alanine (+)
Fructose (+)	Sucrose (+)
Myo-inositol phosphate (−)	Pipecolic acid (−)
Methionine (−)	cis-Aconitic acid (−)
2-Oleoylglycerol (−)	Citric acid (−)
Glutamine (−)	Tetrahydroxybenzene (−)
Glyceric acid (+)	γ-Tocopherol (−)
Pipecolic acid (+)	δ-Tocopherol (−)
Hydroxyquinolinic acid (+)	Isocitric acid (−)

**Table 2:** Tentatively identified compounds with the highest loadings in the first two principal components derived from PLS-DA of metabolite profiles of four *Fucus* species. The vertical order in the table is according to the absolute values of the loadings—for each component, the compound with the highest value is in the first line. Signs in the brackets mean positive or negative values.

Component 1	Component 2
Carbohydrate RI 2241 (−)	Sucrose (+)
Leucine (+)	Isocitric lactone (+)
Polyol RI 2841 (−)	4-Hydroxybutyric acid (+)
Methionine (+)	Proline (+)
Threonine (+)	Hydroxypyrimidine derivative (+)
Urea (−)	2-Methylserine (+)
Threonic acid-1,4-lactone (−)	Succinic acid (+)
Dehydroascorbic acid dimer (−)	Malic acid (+)
Difucol (−)	Nicotinic acid (+)
3-Hydroxybutyric acid (+)	Glyceric acid (+)
Carbohydrate RI 2064 (+)	Diphlorethol (−)
α-Aminoadipic acid (+)	Threitol (+)
Carbohydrate RI 3475 (+)	Carbohydrate RI 2143 (+)
Glutamine (+)	Glycerol (+)
Palmitelaidic acid (+)	Glutamine (−)
Cytosine (+)	Fumaric acid (+)
Isoleucine (+)	Isocitric acid (+)
Polyol RI 2082 (−)	Fructose (+)
Eicosenoic acid (+)	Erythritol (+)
Eicosapentaenoic acid (+)	myo-Inositol-phosphate (−)
Aspartic acid (+)	Fucose (+)
Phloroglucinic acid derivative (−)	Alanine (−)
Mannitol (−)	Cholesterol (−)
Phloroglucinol trimer RI 3618 (−)	Linoleic acid (−)
Tetrahydroxybenzene (−)	Homogentisic acid (−)

**Table 3:** Low-molecular-weight metabolites, specifically abundant in the thalli of different fucalean algae.

Algae	5 < FC ^1^ < 10	FC > 10
*Fucus* spp.	Sucrose Disaccharide RI 2722 Trisaccharide RI 3607 Trisaccharide RI 3597 *myo*-Inositol phosphate Mannitol phosphate Glucose-6-phosphate Pyroglutamic acid Methionine S-Methylcysteine 4-Hydroxybutyric acid Cytosine Phloroglucinic acid derivative	Trisaccharide RI 3440 Asparagine Glutamine 2-Methylserine α-Ketoglutaric acid 3-Hydroxybutyric acid Hypoxanthine Difucol Phloroglucinol trimer RI 3618 Homogentisic acid
*F. vesiculosus*	Sucrose Carbohydrate RI 2241 2-Methylserine Tyrosine Squalene Difucol Phloroglucinic acid derivative	Trisaccharide RI 3440 Phloroglucinol trimer RI 3587 Phloroglucinol trimer RI 3618
*F. serratus*	myo-Inositol phosphate Glutamine	Homogentisic acid
*F. spiralis*	Carbohydrate RI 2064 Succinic acid Malic acid 4-Hydroxybutyric acid Cytosine Phytol	γ-Aminobutyric acid α-Ketoglutaric acid 3-Hydroxybutyric acid Nicotinic acid Hypoxanthine
*F. distichus* subsp. *edentatus*	Trisaccharide RI 3597 Dehydroascorbic acid dimer	–
*Ascophyllum nodosum*	Polyol RI 2006 Carbohydrate RI 3079 Carbohydrate RI 2474 γ-Tocopherol	Polyol RI 3547 Polyol RI 2841 Polyol RI 2834 Disaccharide RI 2879 Dihydroxyphenylalanine α-Tocopherol derivative
*Pelvetia canaliculata*	Glycerol Carbohydrate RI 2106 Isocitric lactone Isocitric acid Citric acid Citramalic acid Tyrosol Hydroxyquinolinic acid	Volemitol Polyol RI 2141 Polyol RI 2850 Carbohydrate RI 3167 Carbohydrate RI 2288 1-Aminocyclopropanecarboxylic acid Pipecolic acid Urea
*Sargassum muticum*	Mannose Isofloridoside Erythronic acid Proline Isoleucine Valine Fumaric acid 2-Isopropylmalic acid Palmitelaidic acid Phenethylamine	Polyol RI 2096 Disaccharide RI 3023 Glyceric acid Amine RI 1416 Adenosine

^1^ FC—fold change; significant (*p* < 0.05, FDR adjusted) difference between the relative content of metabolites in representatives of one algal species or genus (for *Fucus* spp.) and the average content in representatives of all other studied species.

**Table 4:** Typical habitats of fucalean algae at sampling sites.

Species	Sampling Site	Typical Habitat
*Sargassum muticum*	Helgoland Isl. (North Sea)	Shallow subtidal—low-intertidal pools, no desiccation during low tide
*Fucus serratus*	Helgoland Isl. (North Sea)	Shallow subtidal—low-intertidal
*F. distichus* subsp. *edentatus*	Keret Archipel. (White Sea)	Shallow subtidal—low-intertidal (the upper boundary is slightly higher, compared to *F. serratus*)
*Ascophyllum nodosum*	Helgoland Isl. (North Sea)	Low-intertidal—mid-intertidal, wave-protected sites
*F. vesiculosus*	Helgoland Isl. (North Sea)	Low-intertidal—mid-intertidal (the upper boundary is slightly higher, compared to *A. nodosum*); both wave-protected and exposed sites
*F. spiralis*	Helgoland Isl. (North Sea)	High-intertidal, sheltered or moderately exposed sites
*Pelvetia canaliculata*	Keret Archipel. (White Sea)	High-intertidal, wave-exposed rocks at the uppermost boundary of the intertidal zone

## Data Availability

The datasets generated and/or analyzed in this study are available from the corresponding authors upon reasonable request.

## Supplementary Materials

The following supporting information can be downloaded at: https://www.mdpi.com/article/10.3390/md24070227/s1, Table S1: Low molecular weight metabolites detected by GC-MS analysis in methanolic extracts of seven algal species of the order Fucales; Figure S1: Sample scores for the first three components derived from PLS-DA of the relative metabolite concentrations in the thalli of seven representatives of the order Fucales; Figure S2: Sample scores for the first three components derived from PLS-DA of the relative metabolite concentrations in the thalli of four representatives of the genus Fucus; Figure S3: Heatmap showing the metabolite profiles of seven brown algae of the order Fucales; Figure S4: Heatmap showing the profiles of phenolic metabolites in seven brown algae of the order Fucales; Figure S5: An example of a dominant species-specific metabolite, polyol RI 3547, which could be detected only in samples of *Ascophyllum nodosum*; Figure S6: GC-MS information supporting the annotation of isofloridoside (a-D-galactopyranosyl-(1-1)-glycerol, RT 31.78, RI 2327.4) in the samples of *Sargassum muticum*; Figure S7: Sample scores for the first two components derived from PLS-DA of the relative concentrations of phenolic metabolites in the thalli of seven representatives of the order Fucales.

## Abbreviations

The following abbreviations are used in this manuscript:

GC-MS	Gas Chromatography–Mass Spectrometry
DOPA	3,4-Dihydroxyphenylalanine
DW	Dry Weight
4-HPA	4-Hydroxyphenylacetic Acid
PLS-DA	Partial Least Squares Discriminant Analysis
RI	Retention Index
RT	Retention Time
SA	Shikimic Acid
TCA	Tricarboxylic Acid
TIC	Total Ion Current
TMS	Trimethylsilyl Group
