# Supplementary material for: GC-MS-Based Metabolomics Provides Insights into the Biochemical Peculiarity of Seven Brown Algal Species of the Order Fucales
Source: Mar Drugs. 2026 Jun 29;24(7):227. doi: 10.3390/md24070227 (PMC13412289; doi:10.3390/md24070227)
Supplement: Supplementary file 1 [file marinedrugs-24-00227-s001.zip › marinedrugs-4347857-supplementary.pdf]

marine drugs

MDPI

Supplementary materials

GC-MS-Based Metabolomics Provides Insights into the Biochemical Peculiarity of Seven Brown Algal Species of the Order Fucales

Elena Tarakhovskaya [1,2,*], Ekaterina Gulk [1], Bochao Yang [3,4], Paula Schliebe [3,5], Susan Billig [3] and Claudia Wiesner [3,6,*]

[1] Department of Plant Physiology and Biochemistry, Faculty of Biology, Saint Petersburg State University, Saint Petersburg 199034, Russia; kategulk@gmail.com
[2] Vavilov Institute of General Genetics, Saint Petersburg Branch, Russian Academy of Science, Saint Petersburg 199034, Russia
[3] Institute of Analytical Chemistry, Faculty of Chemistry, Leipzig University, 04103 Leipzig, Germany; yangbochao54@gmail.com (B.Y.); paula.schliebe@bbz.uni-leipzig.de (P.S.); billig@uni-leipzig.de (S.B.)
[4] Biotechnological-Biomedical Centre (BBZ), Leipzig University, 04103 Leipzig, Germany
[5] Guest Science Programme, Biologische Anstalt Helgoland, Alfred-Wegener-Institut Helmholtz-Zentrum für Polar- und Meeresforschung, 27498 Helgoland, Germany
[6] German Centre for Integrative Biodiversity Research (iDiv) Halle-Jena-Leipzig, 04103 Leipzig, Germany
* Correspondence: elena.tarakhovskaya@gmail.com (E.T.); birkemeyer@chemie.uni-leipzig.de (C.W.)

Table S1. Low molecular weight metabolites detected by GC-MS analysis in methanolic extracts of seven algal species of the order Fucales (*Fucus vesiculosus*, *F. serratus*, *F. spiralis*, *F. distichus* subsp. *edentatus*, *Ascophyllum nodosum*, *Pelvetia canaliculata*, *Sargassum muticum*). Metabolites of phenolic and aromatic nature associated (or putatively associated) with the metabolism of phlorotannins and those associated with phenylalanine/tyrosine metabolism are marked in red or blue font, respectively. The rightmost column gives an indication of the confidence of the tentative assignments: 1 ASL – confirmed by an inhouse library of authentic standards; 2 NIST (reverse match factor) or 2 GMD (match factor) - tentative structural assignment by spectral matching against NIST (National Institute of Standards and Technology) or GMD (Golm Metabolome Database) also considering available RI data; 3 - tentative assignment to a chemical class by manual spectral interpretation based on spectral similarity with reference mass spectra from the library of the same type; 4 - tentative assignment of expected compounds based on information from scientific literature, expected diagnostic ions and retention time range with confirmation by manual spectral interpretation / fragmentation to a different level of detail, including class assignment based on spectral similarity with library compounds of the same type; 5 – labeled as unknown with a speculative class assignment with lower confidence compared to level 4. Particularly the last two levels incorporate a comparably lower level of reliability for the given structural assignment. For compounds at level 1, level 2 is added enabling comparison with the most common commercially available mass spectral library NIST where available.

No.	Metabolite	Derivative	RT (min)	RI	Quant *m/z* ± 0.5	Confidence level of tentative assignment
1	Pyruvic acid	1MEOX, 1TMS	8.68	1019.3	174	1 ASL, 2 NIST (966)
2	Lactic acid	2TMS	8.87	1028.8	117	1 ASL, 2 NIST (957)
3	Glycolic acid	2TMS	9.19	1045.3	177	1 ASL, 2 NIST (861)
4	Butylamine	2TMS	9.53	1063.1	174	2 NIST (867)

5	Alanine	2TMS	9.72	1073.2	116	1 ASL, 2 NIST (948)
6	3-Hydroxybutyric acid	2TMS	10.9	1134.5	191	2 NIST (960)
7	2-Aminobutyric acid	2TMS	11.12	1145.9	130	2 NIST (921)
8	Unknown RI1174	–	11.66	1174	221	–
9	1-Aminocyclopropane-carboxylic acid	2TMS	11.9	1186.1	202	2 NIST (875)
10	Valine	2TMS	12.07	1194.8	144	1 ASL, 2 NIST (950)
11	4-Hydroxybutyric acid	2TMS	12.54	1219.6	233	2 NIST (924)
12	Unknown RI1221	–	12.57	1221.2	248	–
13	Urea	2TMS	12.66	1225.8	189	1 ASL, 2 NIST (879)
14	Unknown RI1228	–	12.7	1227.7	210	–
15	Benzoic acid	1TMS	12.84	1234.8	179	1 ASL, 2 NIST (787)
16	Serine	2TMS	13.03	1244.7	116	1 ASL, 2 NIST (680)
17	Ethanolamine	3TMS	13.14	1250.5	174	3 NIST (953)
18	Phosphoric acid	3TMS	13.24	1256	299	1 ASL, 2 NIST (911)
19	Leucine	2TMS	13.26	1256.8	158	1 ASL, 2 NIST (946)
20	Glycerol	3TMS	13.31	1259.7	205	1 ASL, 2 NIST (960)
21	Isoleucine	2TMS	13.72	1280.8	158	1 ASL, 2 NIST (944)
22	Proline	2TMS	13.83	1286.4	142	1 ASL, 2 NIST (940)
23	Nicotinic acid	1TMS	13.85	1287.4	180	1 ASL, 2 NIST (940)
24	Glycine	3TMS	13.99	1294.5	174	1 ASL, 2 NIST (944)
25	Unknown RI1296	–	14.02	1296.5	254	–
26	Succinic acid	2TMS	14.22	1306.8	247	1 ASL, 2 NIST (956)
27	Unknown RI1313	–	14.35	1313.3	183	–
28	Unknown RI1319	–	14.46	1319.1	196	–
29	Glyceric acid	3TMS	14.54	1323.2	189	1 ASL, 2 NIST (924)
30	Uracil	2TMS	14.67	1330.2	241	1 ASL, 2 NIST (939)
31	Itaconic acid	2TMS	14.82	1338.1	259	1 ASL, 2 NIST (708)
32	Pyrrole-2-carboxylic acid	2TMS	14.95	1344.5	240	2 NIST (765)
33	Fumaric acid	2TMS	15.01	1347.7	245	1 ASL, 2 NIST (926)
34	Alanine	3TMS	15.11	1351.8	188	1 ASL, 2 NIST (819)

35	Serine	3TMS	15.17	1355.8	204	1 ASL, 2 NIST (910)
36	Pipecolic acid	2TMS	15.23	1359.3	156	2 NIST (855)
37	Nonanoic acid	1TMS	15.26	1360.6	215	1 ASL, 2 NIST (553)
38	Unknown RI1361	–	15.27	1361.1	224	–
39	3-Cyanoalanine	2TMS	15.44	1370.2	141	2 NIST (889)
40	Threonic acid-1,4-lactone	2TMS	15.49	1372.5	247	2 NIST (775)
41	2-Methylserine	2TMS	15.5	1373	130	2 GMD (789)
42	Threonine	3TMS	15.7	1383.5	218	1 ASL, 2 NIST (942)
43	O-Acetylserine	2TMS	15.93	1395.3	174	2 NIST (770)
44	Unknown RI1398	–	15.97	1397.7	264	–
45	Thymine	2TMS	16.01	1399.5	255	2 GMD (933)
46	Glutaric acid	2TMS	16.17	1407.7	261	1 ASL, 2 NIST (814)
47	Amine RI1416	–	16.32	1415.8	174	5
48	S-Methylcysteine	2TMS	16.46	1422.9	218	2 GMD (874)
49	β-Alanine	3TMS	16.59	1429.6	174	1 ASL, 2 NIST (948)
50	3,4-Dihydroxybutyric acid	3TMS	16.74	1438	233	2 NIST (817)
51	Homoserine	3TMS	17.06	1454	218	1 ASL, 2 NIST (780)
52	Unknown RI1459	–	17.15	1458.7	350	–
53	Decanoic acid	1TMS	17.28	1465.6	229	2 NIST (815)
54	Aspartic acid deriv.	3TMS	17.46	1474.7	232	3 NIST (808)
55	Citramalic acid	2TMS	17.5	1477.1	247	2 NIST (784)
56	Unknown RI1479	–	17.55	1479.5	155	–
57	Unknown RI1480	–	17.57	1480	255	–
58	Erythrulose	1MEOX, 3TMS	17.7	1487.2	262	1 ASL, 2 NIST (760)
59	Aspartic acid deriv.	3TMS	17.73	1489	232	3 NIST (790)
60	Malic acid	3TMS	17.89	1497	233	1 ASL, 2 NIST (920)
61	Unknown RI1507	–	18.07	1506.7	305	–
62	Threitol	4TMS	18.11	1508.9	217	2 NIST (720)
63	Asparagine (−H_2O)	2TMS	18.17	1511.8	243	2 GMD (869)
64	Erythritol	4TMS	18.24	1515.6	217	1 ASL, 2 NIST (867)

65	Methionine	2TMS	18.47	1527.1	176	1 ASL, 2 NIST (863)
66	Aspartic acid	3TMS	18.5	1528.8	232	1 ASL, 2 NIST (885)
67	Pyroglutamic acid	2TMS	18.51	1529.4	156	1 ASL, 2 NIST (941)
68	Cytosine	2TMS	18.57	1532.5	240	2 NIST (785)
69	Unknown RI1534	–	18.59	1533.7	305	–
70	Unknown RI1536	–	18.64	1536.4	183	–
71	4-Aminobutyric acid	3TMS	18.67	1537.7	174	2 NIST (833)
72	Glutamic acid	2TMS	18.81	1545.2	174	1 ASL, 2 NIST (846)
73	Pyrogallol	3TMS	18.84	1546.3	239	2 NIST (759)
74	Erythronic acid	4TMS	19.28	1569.3	292	1 ASL, 2 NIST (912)
75	Phenethylamine	2TMS	19.35	1573.1	174	2 NIST (825)
76	Unknown RI1577	–	19.43	1577.1	202	–
77	4-Hydroxyphenylethanol (tyrosol)	2TMS	19.52	1581.9	179	2 NIST (711)
78	2-Isopropylmalic acid	3TMS	19.53	1582.4	275	2 NIST (730)
79	α-Ketoglutaric acid	1MEOX, 2TMS	19.64	1588.2	198	1 ASL, 2 NIST (886)
80	Unknown RI1593	–	19.73	1592.8	142	–
81	Asparagine deriv.	–	19.85	1599.5	218	2 GMD (700)
82	Glutamine (−H_2O)	3TMS	19.88	1601.4	227	2 GMD (805)
83	Hydroxyquinol	3TMS	20.06	1612.3	342	3 NIST (831)
84	Hydroxypipecolic acid	3TMS	20.07	1612.7	244	2 NIST (733)
85	Glutamic acid	3TMS	20.5	1638.2	246	1 ASL, 2 NIST (951)
86	Phenylalanine	2TMS	20.54	1640.4	218	1 ASL, 2 NIST (923)
87	p-Hydroxybenzoic acid	2TMS	20.67	1647.8	267	2 NIST (653)
88	Unknown RI1653	–	20.75	1652.6	201	–
89	4-Hydroxyphenylacetic acid, 4HPA	2TMS	20.88	1660	179	2 NIST (851)
90	Phloroglucinol	3TMS	20.92	1662.7	342	1 ASL, 2 NIST (909)
91	Unknown RI1683	–	21.26	1682.8	224	–
92	Arabinose	1MEOX, 4TMS	21.27	1683.3	103	1 ASL, 2 NIST (715)
93	Unknown RI1686 (benzene-triol like compound with increased *m*/*z* 311)	–	21.33	1686	342	4 NIST (665)

94	2,4,5-Trihydroxypentanoic acid	–	21.35	1687.9	245	2 NIST (682)
95	Hydroxypyrimidine deriv.	3TMS	21.42	1692	358	3 NIST (715)
96	Asparagine	3TMS	21.43	1692.9	116	2 NIST (947)
97	Ribose	1MEOX, 4TMS	21.55	1699.8	103	1 ASL, 2 NIST (935)
98	α-Aminoadipic acid	3TMS	21.72	1710.2	260	2 NIST (726)
99	Pentitol RI1718	–	21.86	1718.1	319	4
100	Unknown RI1721	–	21.91	1721.2	275	–
101	Xylitol	5TMS	22.06	1729.7	217	1 ASL, 2 GMD (786)
102	Unknown RI1732	–	22.09	1731.8	304	–
103	Isocitric lactone	2TMS	22.15	1735.2	129	2 NIST (765)
104	Dihydroxybisnaphthol	2TMS	22.21	1739	430	3 NIST (776)
105	Arabitol	5TMS	22.31	1744.8	217	1 ASL, 2 NIST (888)
106	Fucose	1MEOX, 4TMS	22.36	1747.6	117	1 ASL, 2 NIST (640)
107	Glycerol-2-phosphate	4TMS	22.48	1754.9	243	2 NIST (722)
108	Glutamine	4TMS	22.52	1756.9	227	1 ASL, 2 NIST (910)
109	Unknown RI1762	–	22.6	1762	392	–
110	Unknown RI1766	–	22.67	1765.8	289	–
111	Pyrimidinecarboxylic acid deriv.	3TMS	22.77	1771.6	254	3 NIST (806)
112	cis-Aconitic acid	3TMS	22.9	1779.2	229	1 ASL, 2 NIST (791)
113	Tetrahydroxybenzene	4TMS	23.04	1787.8	430	4
114	Unknown RI1789	–	23.06	1788.8	392	–
115	Glycerol-3-phosphate	4TMS	23.08	1789.9	357	1 ASL, 2 NIST (941)
116	Lyxonic acid	5TMS	23.28	1802	292	3 NIST (774)
117	Glutamine	3TMS	23.29	1802.5	156	1 ASL, 2 NIST (943)
118	2,5-Dihydroxybenzoic acid (gentisic acid)	3TMS	23.32	1804.5	355	3 NIST (879)
119	Unknown RI1807	–	23.36	1806.6	319	–
120	Unknown RI1824	–	23.67	1824.5	392	–
121	Azelaic acid	2TMS	23.72	1827.5	317	1 ASL, 2 NIST (748)
122	Hypoxanthine	2TMS	23.85	1835.2	265	2 NIST (905)

123	Citric acid	4TMS	24.06	1847.6	257	1 ASL, 2 NIST (955)
124	Isocitric acid	4TMS	24.12	1851.4	245	2 NIST (700)
125	Homogentisic acid	3TMS	24.34	1864.1	384	1 ASL, 2 NIST (880)
126	Neophytadiene	–	24.52	1875	123	3 NIST (849)
127	Dehydroascorbic acid dimer	–	24.55	1876.9	173	2 NIST (761)
128	Unknown RI1880	–	24.61	1880.3	259	–
129	Myristic acid	1TMS	24.63	1881.3	285	2 NIST (956)
130	Adenine	2TMS	24.9	1897.2	264	1 ASL, 2 NIST (911)
131	Fatty alcohol RI1899	–	24.94	1899.5	123	3 NIST (857)
132	Sorbose peak 1	1MEOX, 5TMS	24.94	1899.6	307	1 ASL, 2 NIST (709)
133	Fructose peak 1	1MEOX, 5TMS	24.97	1901.2	307	1 ASL, 2 NIST (803)
134	Sorbose peak 2	1MEOX, 5TMS	25.05	1906	307	1 ASL, 2 NIST (709)
135	Fructose peak 2	1MEOX, 5TMS	25.12	1910	307	1 ASL, 2 NIST (755)
136	Mannose	1MeOX, 5TMS	25.21	1915.3	319	1 ASL, 2 NIST (760)
137	Galactose	1MeOX, 5TMS	25.25	1917.6	319	1 ASL, 2 NIST (917)
138	Unknown RI 1919 (Phytol acetate)	–	25.26	1918.5	123	5 NIST (902)
139	Tyrosine	2TMS	25.30	1920.6	179	2 NIST (859)
140	Glucose peak 1	1MeOX, 5TMS	25.35	1923.9	319	1 ASL, 2 NIST (942)
141	Tyramine	3TMS	25.64	1941	174	2 NIST (881)
142	Glucose peak 2	1MeOX, 5TMS	25.67	1942.1	319	1 ASL, 2 NIST (839)
143	Galacturonic acid	1MeOX, 5TMS	25.74	1946.6	333	1 ASL, 2 NIST (896)
144	Mannitol	6TMS	25.9	1955.9	421	1 ASL, 2 NIST (956)
145	Dihydroxynaphthoic acid (*coelution with mannitol*)	3TMS	26.0	1957	420	4
146	Tyrosine	3TMS	26.08	1966.5	218	1 ASL, 2 NIST (891)
147	Sorbitol	6TMS	26.13	1969.5	319	1 ASL, 2 NIST (847)
148	Ascorbic acid	4TMS	26.6	1997.1	332	1 ASL, 2 NIST (634)

149	Polyol RI2006	–	26.74	2005.6	305	3
150	Phloroglucinic acid	4TMS	26.88	2013.6	443	1 ASL, 2 NIST (859)
151	Carbohydrate RI2028	–	27.13	2028.4	175	3
152	Unknown RI2037	–	27.28	2037.2	271	–
153	Gluconic acid	6TMS	27.29	2037.3	333	1 ASL, 2 NIST (879)
154	Hydroxyquinolic acid (trihydroxybenzoic acid)	4TMS	27.3	2038	443	3 NIST (736)
155	Xanthine	3TMS	27.38	2043.2	353	2 NIST (824)
156	Unknown RI2046	–	27.43	2046.2	231	–
157	scyllo-Inositol	6TMS	27.48	2049.3	318	2 NIST (734)
158	Palmitelaidic acid	1TMS	27.51	2051.3	311	1 ASL, 2 NIST (953)
159	Carbohydrate RI2064	–	27.74	2064.4	217	3
160	Palmitic acid	1TMS	27.87	2072.4	313	1 ASL, 2 NIST (952)
161	Unknown RI2081	–	28.02	2081.4	175	–
162	Polyol RI2082	–	28.03	2082	217	3
163	Unknown RI2083	–	28.05	2083	261	–
164	Dihydroxyphenylalanine (DOPA)	3TMS	28.15	2088.4	267	2 GMD (872)
165	Polyol RI2096	–	28.27	2095.8	204	3
166	Unknown RI2101	–	28.38	2101	231	–
167	Myo-Inositol	6TMS	28.44	2105.9	318	1 ASL, 2 NIST (943)
168	Dihydroxyanthraquinone	–	28.44	2106	369	3
169	Carbohydrate RI2106	–	28.45	2106.4	259	3
170	Carbohydrate RI2111	–	28.54	2111.4	332	3
171	Unknown RI2113 (tentative triolbenzoate / dihydroxyanthraquinone)	–	28.56	2113	369	5
172	Dihydroxyphenylalanine (DOPA)	4TMS	28.59	2114.8	218	2 NIST (927)
173	Uric acid	4TMS	28.65	2118.1	441	2 NIST (880)
174	Unknown RI2119 (tentative phenolic acid)	–	28.67	2119.2	179	5
175	Unknown RI2135	–	28.97	2135	387	–
176	Unknown RI2140	–	29.02	2139.8	325	–
177	Guanine	3TMS	29.04	2141.1	352	2 NIST (941)
178	Polyol RI2142	–	29.07	2142	259	3

179	Carbohydrate RI2143	–	29.08	2143.4	319	3
180	Carbohydrate RI2149	–	29.17	2148.6	319	3
181	Unknown RI2156	–	29.3	2156.5	292	–
182	Heptadecanoic acid	1TMS	29.36	2160.2	327	2 NIST (818)
183	Unknown RI2161 (tentative triolbenzoate deriv. / dihydroxyanthraquinone)	–	29.38	2160.9	369	5
184	Phytol	1TMS	29.78	2184.5	123	2 NIST (957)
185	Carbohydrate RI2194	–	29.94	2194.2	305	3
186	Unknown RI2197 (tentative eckol)	–	29.98	2196.8	521	5
187	Unknown RI2202 (tentative dihydroxy methylbenzoate deriv.)	–	30.10	2202.1	297	5
188	Glycerophosphoglycerol	5TMS	30.11	2204.1	357	2 NIST (728)
189	Octadecatrienoic acid	1TMS	30.13	2205.2	79	2 NIST (832)
190	Fatty acid deriv.	–	30.21	2209.3	299	5
191	Unknown RI2217 (tentative triolbenzene derivative)	–	30.33	2217.1	342	5
192	Linoleic acid	1TMS	30.35	2218.7	337	2 NIST (869)
193	Volemitol	–	30.44	2223.4	421	4
194	Oleic acid	1TMS	30.47	2225.4	339	2 NIST (932)
195	Carbohydrate RI2241 (Floridoside?)	–	30.73	2241	204	4
196	Stearic acid	1TMS	30.84	2247.4	341	1 ASL, 2 NIST (949)
197	Carbohydrate RI2288	–	31.31	2287.7	204	3
198	Difucol (main peak)	6TMS	31.48	2302.6	682	4
199	Fructose-6-phosphate	1MEOX, 6TMS	31.64	2316.3	315	1 ASL, 2 NIST (624)
200	Isofloridoside	6TMS	31.77	2327.4	337	1 ASL, 2 NIST (895)
201	Glucose-6-phosphate	1MEOX, 6TMS	31.81	2330.8	387	1 ASL, 2 NIST (671)
202	Unknown RI2345 (tentative phenolic acid)	–	31.98	2345	384	5
203	Unknown RI2348	–	32.01	2347.6	292	–
204	Mannitol phosphate	7TMS	32.24	2367	387	2 NIST (861)
205	Diphlorethol	5TMS	32.32	2374.2	610	4
206	Polyol RI2378	–	32.37	2378.4	331	3

207	Arachidonic acid	1TMS	32.53	2392.5	117	2 NIST (957)
208	Unknown RI2395 (tentative phenolic acid)	–	32.56	2395	267	5
209	Eicosapentaenoic acid	1TMS	32.63	2400.4	117	2 NIST (959)
210	Unknown RI2409	–	32.72	2408.8	470	–
211	Unknown RI2411	–	32.75	2411.2	585	–
212	Diphlorethol (main peak)	5TMS	32.87	2421.3	610	4
213	Glycerol-myristate	2TMS	32.92	2425.8	343	2 NIST (722)
214	Unknown RI2430	–	32.97	2430.1	470	–
215	myo-Inositol phosphate	7TMS	33.08	2439.1	318	2 NIST (915)
216	Eicosadienoic acid	1TMS	33.13	2444	365	2 NIST (780)
217	11-Eicosenoic acid	1TMS	33.14	2445	367	2 NIST (801)
218	Unknown RI2451 (tentative triolbenzoate deriv., phlorethol / dihydroxyanthraquinone)	–	33.21	2450.7	369	5
219	Carbohydrate RI2474	–	33.49	2474.3	217,330	3
220	Eicosanoic acid	1TMS	33.56	2479.8	369	2 NIST (802)
221	Uridine	3TMS	33.59	2482.6	259	2 NIST (871)
222	Unknown RI2486	–	33.63	2486.1	435	–
223	Phloroglucinic acid deriv.	–	33.94	2512.7	443	5
224	Unknown RI2515	–	33.96	2514.8	639	–
225	Difucol	6TMS	33.98	2515	682	4
226	Hydroxy-difucol	7TMS	34.06	2522.5	770	4
227	Hydroxy-diphlorethol	6TMS	34.09	2526	698	4
228	Unknown RI2532 (tentative triolbenzoate glycoside)	–	34.16	2531.7	369	5
229	Unknown RI2544 (tentative phenolic acid)	–	34.31	2544.2	355	5
230	Diphlorethol	5TMS	34.35	2547.7	610	4
231	Hydroxy-diphlorethol	6TMS	34.44	2555.4	698	4
232	Unknown RI2556 (tentative triolbenzoate glycoside)	–	34.45	2556.3	369	5
233	Benzenetriolic acid deriv. RI2568	–	34.58	2567.8	443	5
234	Benzenetriolic acid deriv. RI2575	–	34.67	2574.7	443	5

235	Benzenetriolic acid deriv. RI2602	–	34.99	2602.2	443	5
236	2-Palmitoylglycerol	2TMS	35.01	2604.1	313	2 NIST (789)
237	Unknown RI2613 (tentative hydroxylated eckol)	–	35.11	2613	624	5
238	Inosine	4TMS	35.2	2620.6	281	3 NIST (752)
239	Unknown RI2630 (tentative triolbenzoate deriv.)	–	35.31	2630.2	369	5
240	Adenosine	3TMS	35.41	2638.4	230	2 NIST (746)
241	1-Palmitoylglycerol	2TMS	35.43	2639.8	371	2 NIST (850)
242	Unknown RI2654	–	35.6	2654.4	483	–
243	Erucic acid	1TMS	35.68	2661.6	395	2 NIST (761)
244	Difucol	6TMS	35.69	2662	682	4
245	2-deoxyadenosine	3TMS	35.81	2672.2	207	2 GMD (688)
246	Eckol / carmalol	–	35.86	2676.7	536	5
247	Sucrose	8TMS	35.97	2686.6	361	1 ASL, 2 NIST (949)
248	Adenosine	4TMS	35.98	2686.9	236	2 NIST (899)
249	Disaccharide RI2722	–	36.39	2722.1	307	3
250	Polyol RI2734	–	36.53	2733.7	319	3
251	Unknown RI2752 (M^{+*}=665)	–	36.74	2752.1	519	–
252	Disaccharide RI 2757 (Cellobiose ?)	1MEOX, 8TMS	36.8	2757.2	361	3 NIST (924)
253	9-Octadecenoic acid deriv.	–	37.02	2775.5	337	3 NIST (791)
254	2-Oleoylglycerol	2TMS	37.07	2779.8	339	2 NIST (822)
255	Maltose	8TMS	37.17	2788.7	361	1 ASL, 2 NIST (889)
256	Unknown RI2793 (tentative eckol / carmalol)	–	37.22	2793.4	536	5
257	Carbohydrate RI2794	–	37.23	2793.6	333	3
258	Trehalose	8TMS	37.25	2795.7	361	1 ASL, 2 NIST (901)
259	Unknown RI2811	–	37.43	2811	395	–
260	1-Oleoylglycerol	2TMS	37.49	2815.8	397	2 NIST (804)
261	Unknown RI2819	–	37.52	2818.7	375	–
262	Polyol RI2834	–	37.7	2833.7	204	3
263	Polyol RI2841	–	37.78	2841.2	204	3

264	Polyol RI2850 (Lactitol ?)	9TMS	37.89	2850	361	3 NIST (907)
265	Unknown RI2855	–	37.95	2855	392	–
266	cis-15-Tetracosenoic acid	1TMS	38.07	2865.6	423	2 NIST (846)
267	Squalene	–	38.2	2876.6	81	2 NIST (915)
268	Disaccharide RI2879	–	38.22	2878.6	361	3
269	Fatty acid deriv. RI2912	–	38.62	2912.3	103	5
270	Polyol RI2924	–	38.76	2924.4	361	3
271	Fatty acid deriv. RI2949	–	39.04	2948.9	203	5
272	δ-Tocopherol	1TMS	39.13	2956.4	474	2 NIST (915)
273	Carbohydrate RI2961	–	39.19	2961.1	319	3
274	Carbohydrate RI2963	–	39.21	2963.3	375	3
275	Disaccharide RI2968	–	39.27	2967.8	361	3
276	Disaccharide RI3023	–	39.91	3023	361	3
277	Benzene-triol deriv. RI3027	–	39.96	3026.8	342	4
278	β-Tocopherol	1TMS	40.08	3037.2	488	2 NIST (920)
279	γ-Tocopherol	1TMS	40.2	3047.7	488	2 NIST (865)
280	Benzene-triol deriv. RI3050	–	40.23	3049.9	342	4
281	Carbohydrate RI3079	–	40.57	3079.1	343	3
282	Unknown RI3101	–	40.82	3101.1	330	–
283	Unknown RI3138	–	41.26	3138.5	546	–
284	Unknown RI3139 (tentative phenolic acid derivative)	–	41.27	3139	267	5
285	Carbohydrate RI3167	–	41.59	3166.7	204	3
286	α-Tocopherol	1TMS	41.72	3178	502	2 NIST (910)
287	Unknown RI3179	–	41.74	3179.4	475	–
288	Cholesterol	1TMS	41.82	3190	329	2 NIST (734)
289	Sterol RI3222	–	42.24	3222.1	129	–
290	Unknown RI3240	–	42.45	3239.9	221	–
291	Polyol RI3253	–	42.6	3252.6	371	3
292	Sterol RI3272	–	42.82	3271.7	129	5
293	Fatty acid deriv. RI3296	–	43.10	3295.9	257	5

294	α-Tocopherol-hydroquinone	3TMS	43.26	3309	309	3 NIST (758)
295	Unknown RI3319	–	43.38	3319.2	175	–
296	Fucosterol	1TMS	43.84	3358.4	386	2 NIST (946)
297	Unknown RI3369 (tentative dihydroxymethylbenzoate deriv.)	–	44.00	3369.2	297	5
298	Unknown RI3372	–	44.03	3371.8	498	–
299	α-Tocopherol deriv.	–	44.08	3375.6	502	3 NIST (720)
300	Polyol RI3391	–	44.3	3391.4	319	3
301	Unknown RI3424	–	44.75	3424.5	512	–
302	Unknown RI3435	–	44.9	3434.9	512	–
303	Trisaccharide RI3440	–	44.97	3440.1	361	3
304	Phyllodihydroquinone	2TMS	45.05	3446.5	596	2 GMD (795)
305	Unknown RI3456	–	45.19	3456.1	437	–
306	Fatty acid deriv. RI3461	–	45.26	3461.2	285	5
307	Carbohydrate RI3475	–	45.46	3475.4	383	3
308	Unknown RI3478	–	45.49	3477.8	512	–
309	Trisaccharide RI3484 (Cellotriose ?)	1MEOX, 11TMS	45.58	3484.1	204	3 NIST (736)
310	Fatty acid deriv. RI3485	–	45.59	3485.1	343	5
311	Trisaccharide RI3488	–	45.63	3488.1	361	3
312	Unknown RI3497	–	45.76	3497.2	512	–
313	Benzenetriol deriv. RI3509	–	45.92	3509.3	536	4
314	Unknown RI3513	–	45.98	3513.5	481	–
315	Trisaccharide RI3514	–	45.99	3514.1	361	3
316	Unknown RI3519 (tentative triolbenzoate deriv.)	–	46.05	3518.6	647	5
317	Polyol RI3547	–	46.45	3547	361	3
318	Unknown RI3554	–	46.54	3554.2	481	–
319	Phloroglucinol trimer RI3587	–	47	3587.1	342	4
320	Trisaccharide RI3597	–	47.13	3596.8	204	3
321	Trisaccharide RI3607	–	47.28	3607.4	204	3
322	Phloroglucinol trimer RI3618	–	47.42	3617.9	342	4

323	Trisaccharide RI3646	–	47.81	3645.9	204	3
324	Fatty acid deriv. RI3762	–	49.41	3762.1	357	5
325	Fatty acid deriv. RI3769	–	49.51	3769.1	385	5
326	Unknown RI3803 (tentative benzenetriol deriv.)	–	49.98	3802.9	342	5
327	Fatty acid deriv. RI3803	–	49.99	3803.5	343	5
328	Fatty acid deriv. RI3808	–	50.05	3808.4	277	5
329	Unknown RI3834 (tentative triolbenzoate deriv.)	–	50.41	3834.4	369	5
330	Trisaccharide RI3868	–	50.88	3868.3	204	3
331	Fatty acid deriv. RI3995	–	52.63	3995.5	129	5
332	Unknown RI4007	–	52.8	4007.5	157	–
333	Unknown RI4036 (tentative triolbenzoate deriv.)	–	53.2	4036.3	369	5
334	Fatty acid deriv. RI4053	–	53.43	4053.1	371	5

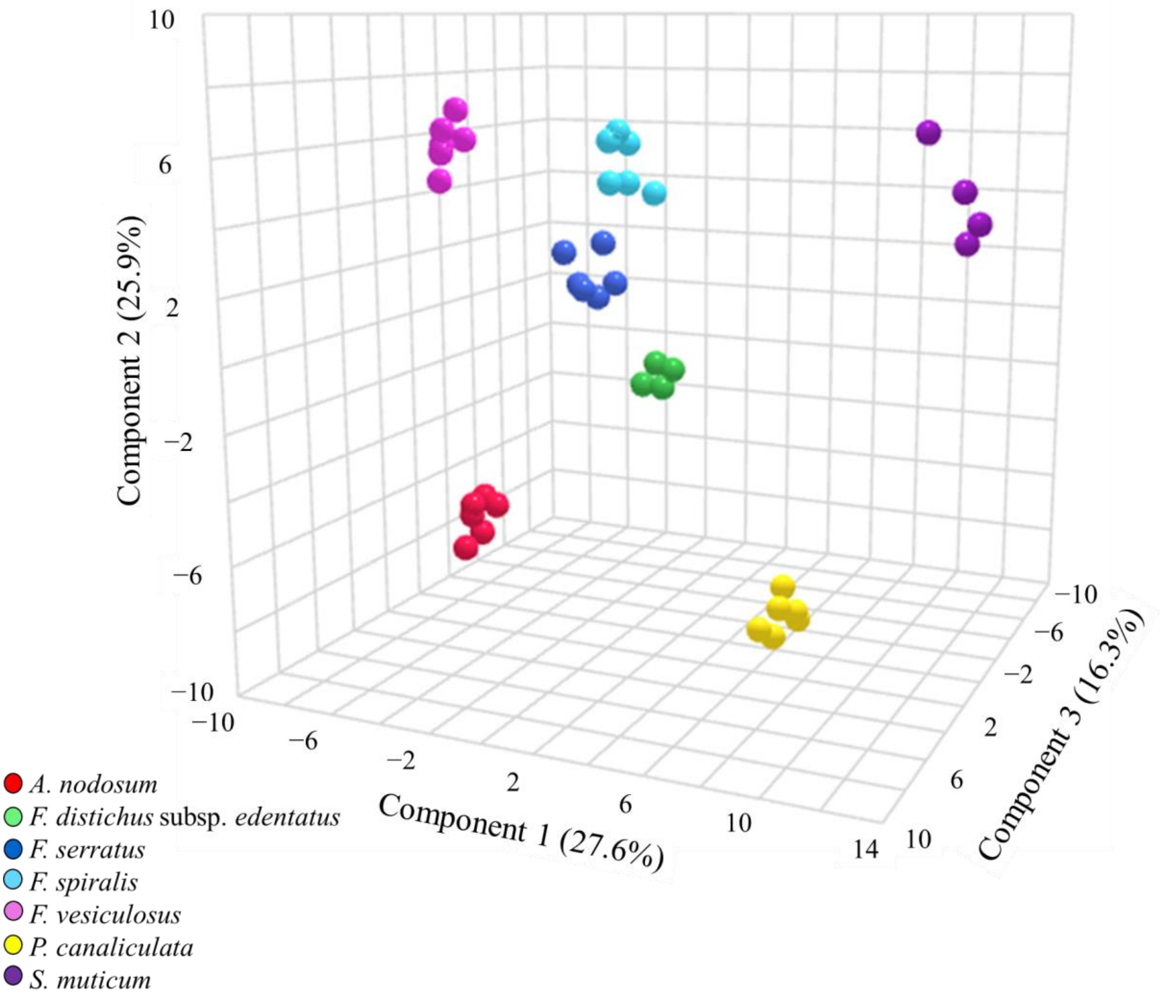

Figure S1. Sample scores for the first three components derived from PLS-DA of the relative metabolite concentrations in the thalli of seven representatives of the order Fucales.

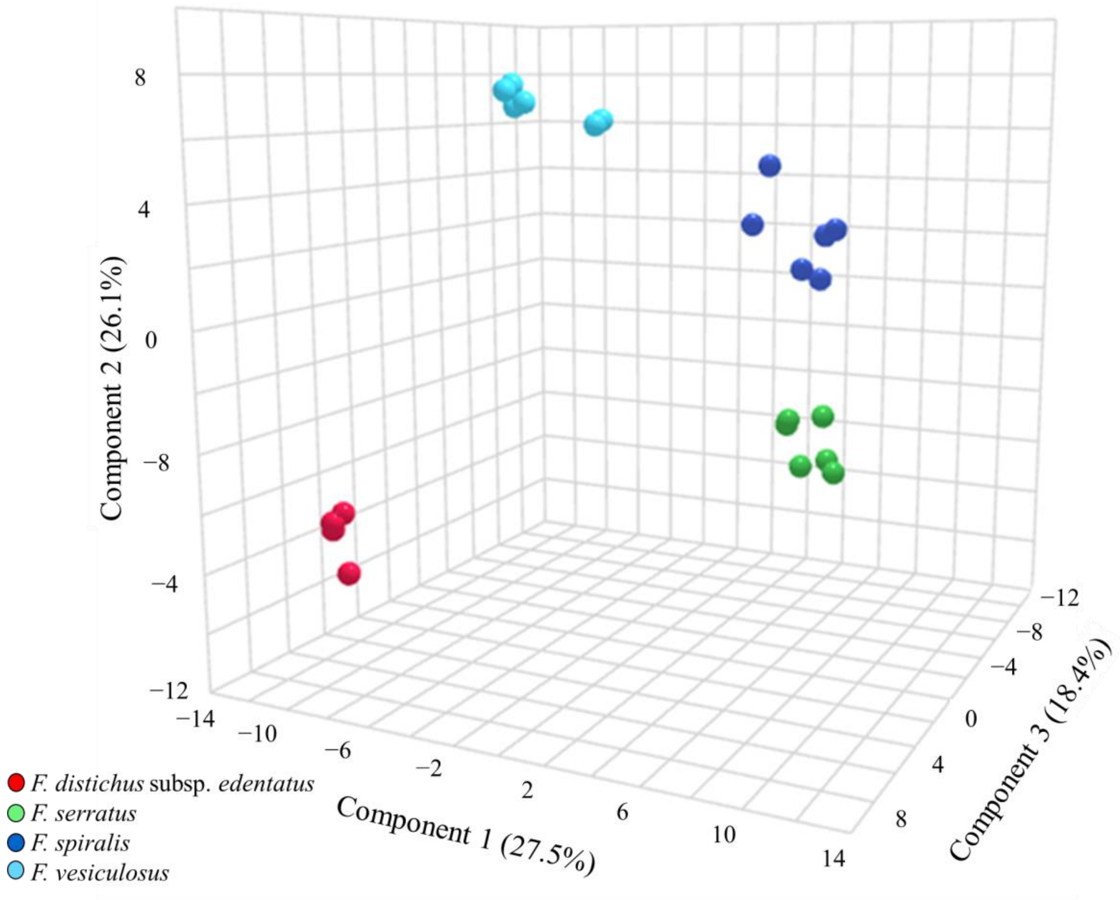

Figure S2. Sample scores for the first three components derived from PLS-DA of the relative metabolite concentrations in the thalli of four representatives of the genus *Fucus*.

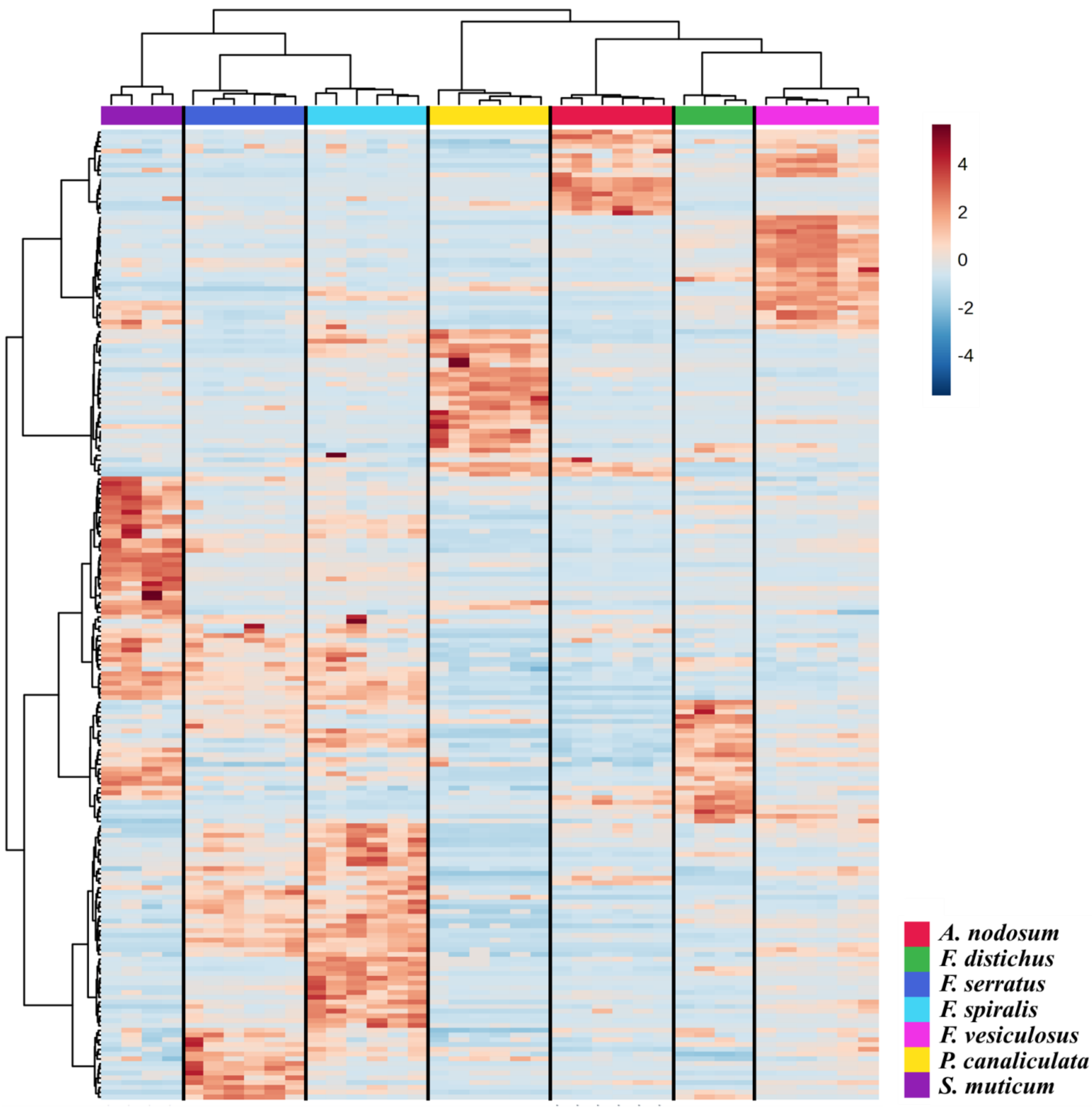

Figure S3. Heatmap showing the metabolite profiles of seven brown algae of the order Fucales (*Fucus vesiculosus*, *F. serratus*, *F. spiralis*, *F. distichus* subsp. *edentatus*, *Ascophyllum nodosum*, *Pelvetia canaliculata*, *Sargassum muticum*). The metabolite list includes all compounds detected by GC-MS approach, both identified and unknown (Table S1). Heatmap was generated using MetaboAnalyst 6.0 (https://www.metaboanalyst.ca).

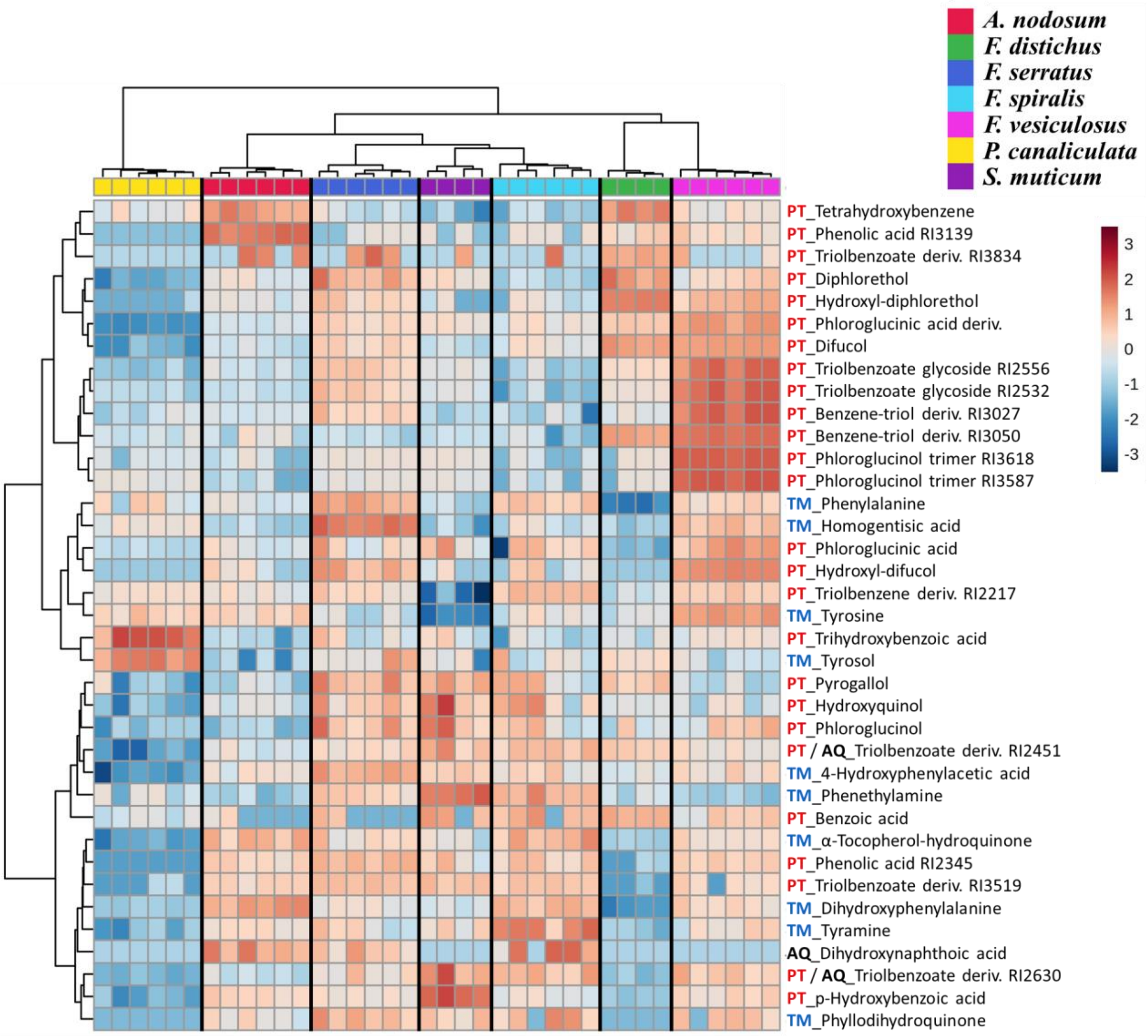

Figure S4. Heatmap showing the profiles of phenolic metabolites in seven brown algae of the order Fucales (*Fucus vesiculosus, F. serratus, F. spiralis, F. distichus* subsp. *edentatus, Ascophyllum nodosum, Pelvetia canaliculata, Sargassum muticum*). **PT** – phlorotannin oligomers and metabolites putatively associated with phlorotannin biosynthesis; **TM** - compounds associated with phenylalanine/tyrosine metabolism; **AQ** – compounds putatively associated with anthraquinone biosynthesis. Heatmap was generated using MetaboAnalyst 6.0 (https://www.metaboanalyst.ca).

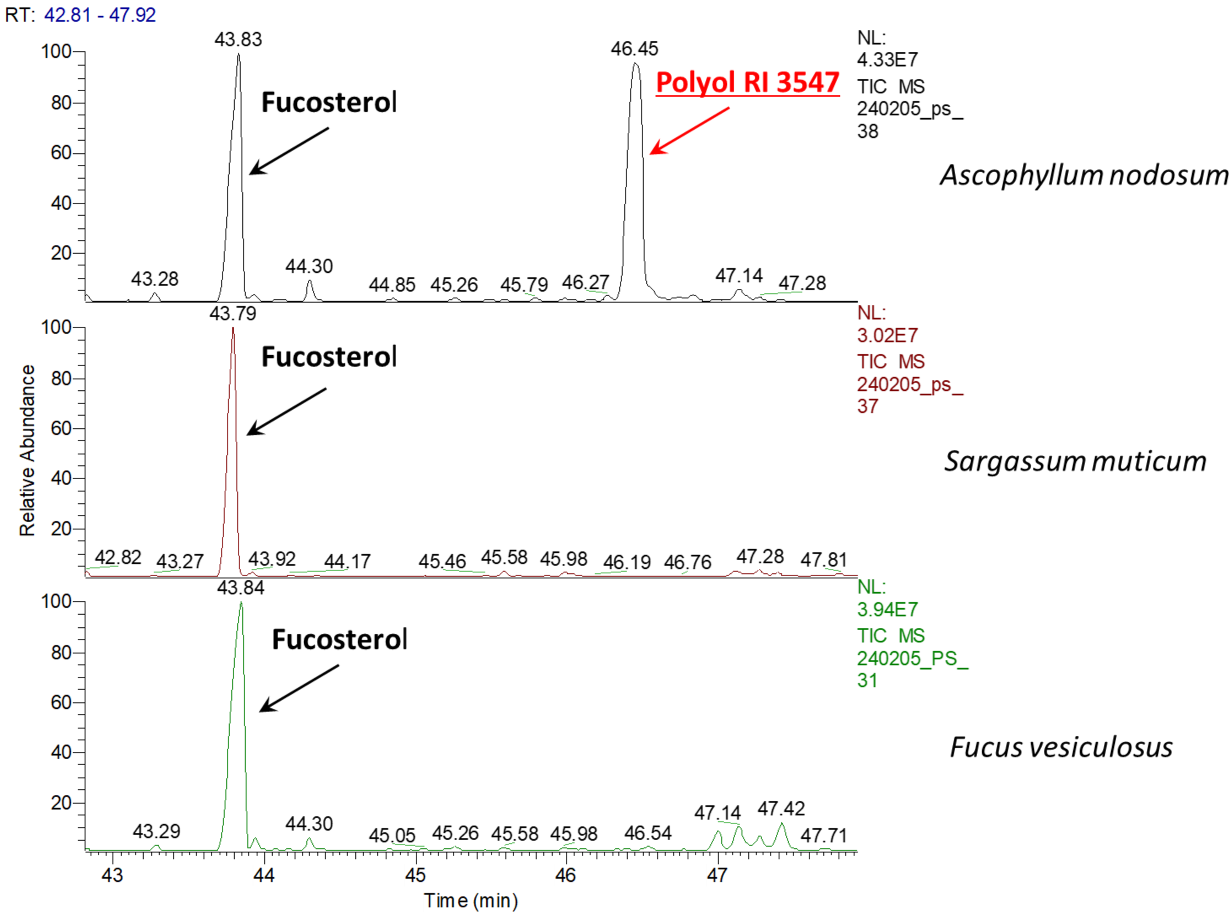

Figure S5. An example of a dominant species-specific metabolite, polyol RI 3547, which could be detected only in samples of *Ascophyllum nodosum*. Total ion chromatograms of *Ascophyllum nodosum*, *Sargassum muticum* and *Fucus vesiculosus* extracts are presented for the RT window of 42.81–47.92 min. Another major peak, corresponding to fucosterol, had similar intensity in all studied algae.

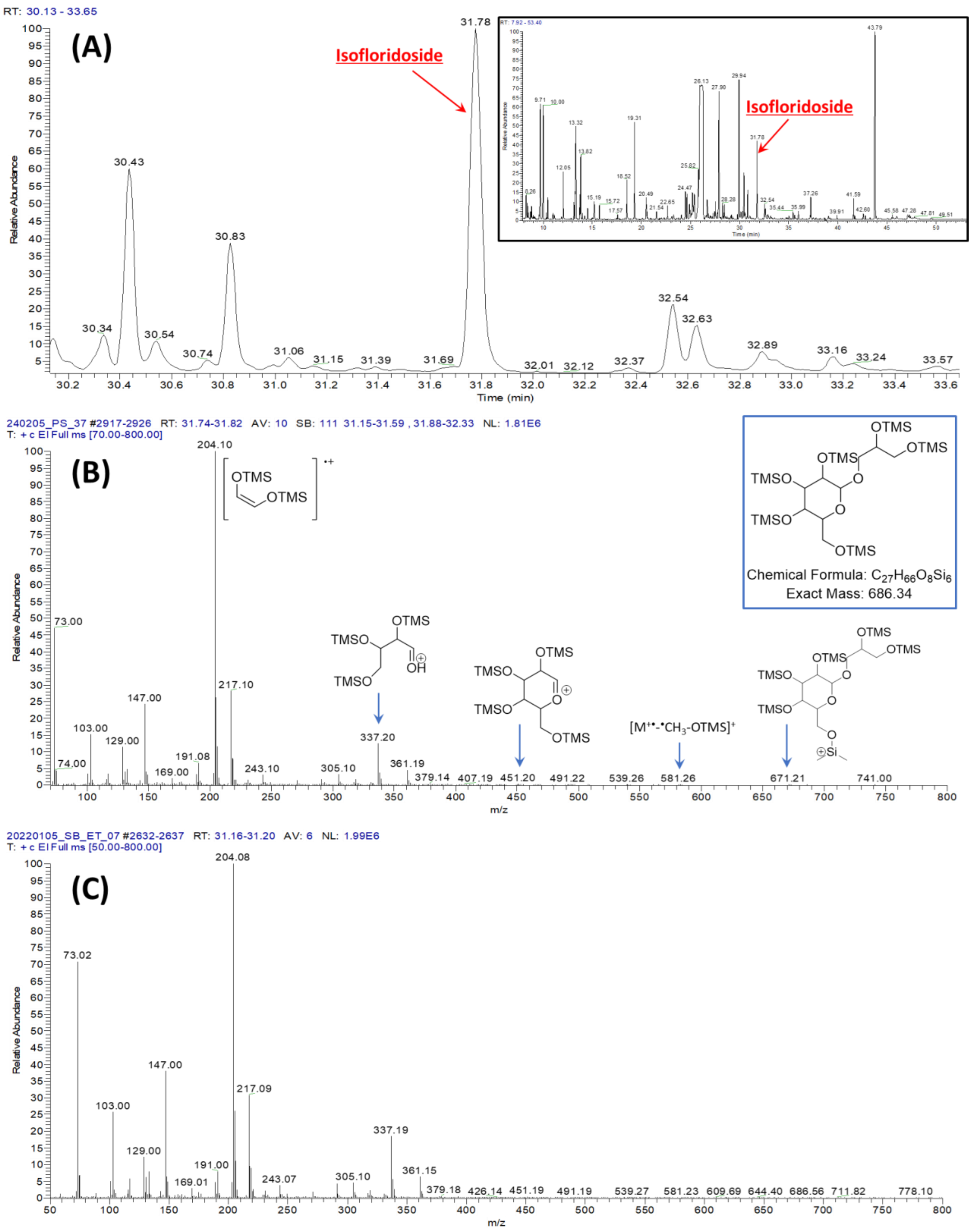

Figure S6. GC-MS information supporting the annotation of isofloridoside (a-D-galactopyranosyl-(1-1)-glycerol, RT 31.78, RI 2327.4) in the samples of *Sargassum muticum*. **A** - Total ion chromatograms of *S. muticum* extract for the RT window of 30.13–33.65 min; the insert presents the full view of the TIC chromatogram, illustrating that the isofloridoside peak is among the dominant ones. **B** - EI mass spectrum of isofloridoside in *S. muticum* extract with characteristic fragmentation pattern. **C** – for comparison, EI mass spectrum of isofloridoside in the extract of the red alga, *Palmaria palmata*. RI of isofloridoside was confirmed by accurate RI information from our inhouse library.

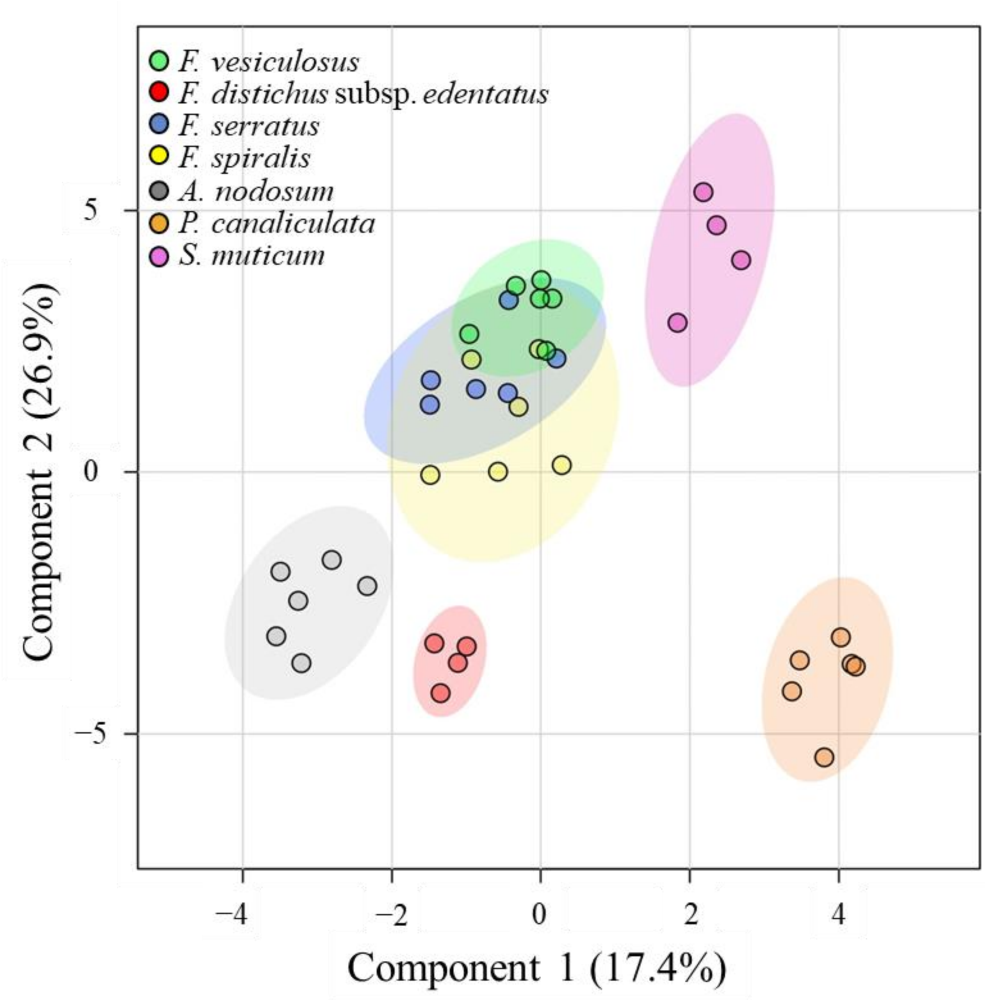

Figure S7. Sample scores for the first two components derived from PLS-DA of the relative concentrations of phenolic metabolites in the thalli of seven representatives of the order Fucales. Analysis was performed using MetaboAnalyst 6.0 (https://www.metaboanalyst.ca).
